# Supplementary material for: A pilot feasibility study of human-centered design for cirrhosis care: Development and pilot testing of SMARTLiver prototype, a FHIR-based clinical decision support system for hepatology
Source: PLOS Digit Health. 2026 Jan 20;5(1):e0000969. doi: 10.1371/journal.pdig.0000969 (PMC12818595; doi:10.1371/journal.pdig.0000969)

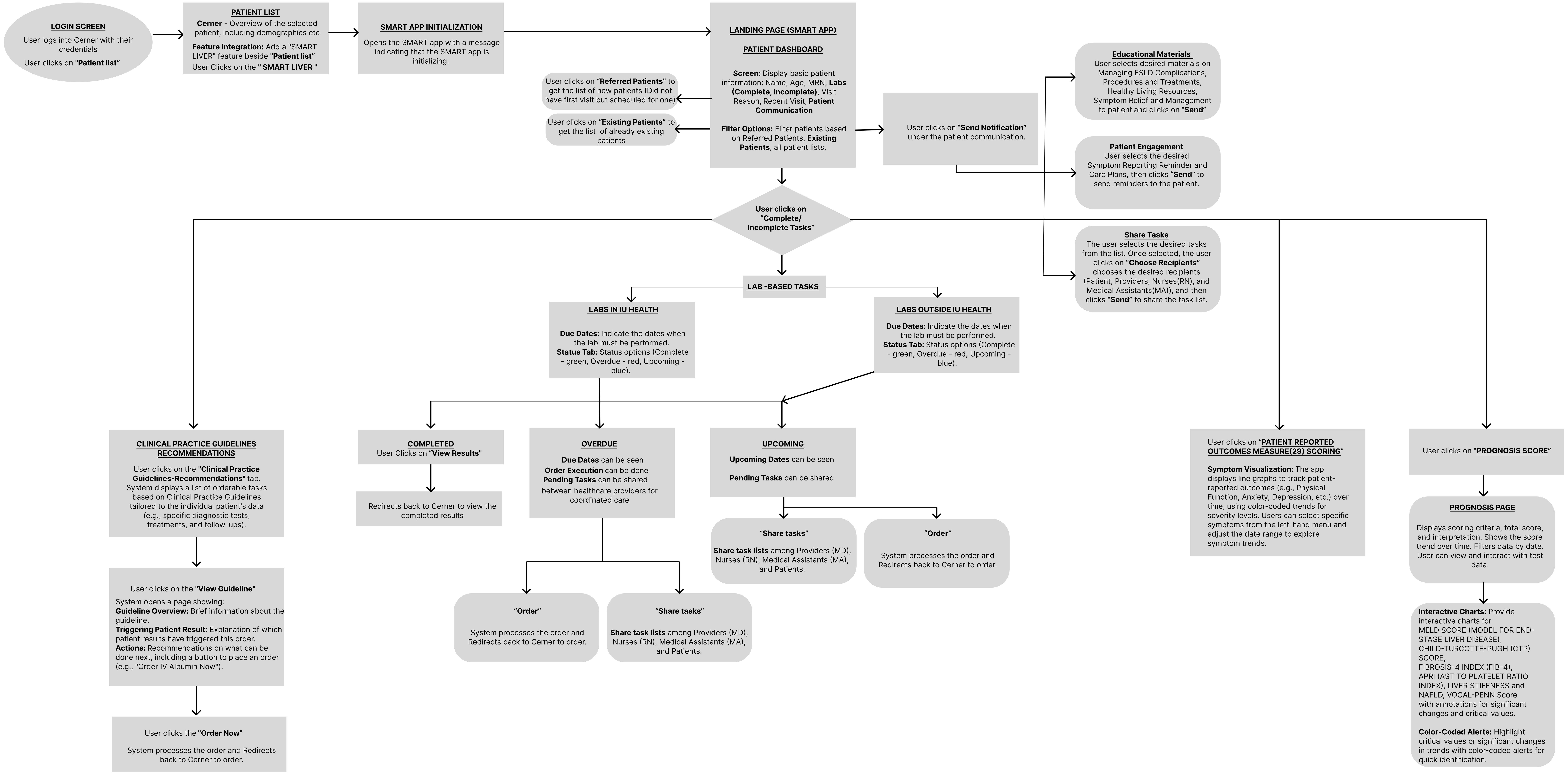
LOGIN SCREEN
User logs into Cerner with their credentials
User clicks on "Patient list"
PATIENT LIST
Cerner - Overview of the selected patient, including demographics etc
Feature Integration: Add a "SMART LIVER" feature beside "Patient list"
User Clicks on the " SMART LIVER "
SMART APP INITIALIZATION
Opens the SMART app with a message indicating that the SMART app is initializing.
LANDING PAGE (SMART APP)
PATIENT DASHBOARD
Screen: Display basic patient information: Name, Age, MRN, Labs (Complete, Incomplete), Visit Reason, Recent Visit, Patient Communication
Filter Options: Filter patients based on Referred Patients, Existing Patients, all patient lists.
User clicks on "Referred Patients" to get the list of new patients (Did not have first visit but scheduled for one)
User clicks on "Existing Patients" to get the list of already existing patients
User clicks on "Send Notification" under the patient communication.
Educational Materials
User selects desired materials on Managing ESLD Complications, Procedures and Treatments, Healthy Living Resources, Symptom Relief and Management to patient and clicks on "Send"
Patient Engagement
User selects the desired Symptom Reporting Reminder and Care Plans, then clicks "Send" to send reminders to the patient.
Share Tasks
The user selects the desired tasks from the list. Once selected, the user clicks on "Choose Recipients" chooses the desired recipients (Patient, Providers, Nurses(RN), and Medical Assistants(MA)), and then clicks "Send" to share the task list.
User clicks on "Complete/ Incomplete Tasks"
LAB -BASED TASKS
LABS IN IU HEALTH
Due Dates: Indicate the dates when the lab must be performed.
Status Tab: Status options (Complete - green, Overdue - red, Upcoming - blue).
LABS OUTSIDE IU HEALTH
Due Dates: Indicate the dates when the lab must be performed.
Status Tab: Status options (Complete - green, Overdue - red, Upcoming - blue).
CLINICAL PRACTICE GUIDELINES RECOMMENDATIONS
User clicks on the "Clinical Practice Guidelines-Recommendations" tab. System displays a list of orderable tasks based on Clinical Practice Guidelines tailored to the individual patient's data (e.g., specific diagnostic tests, treatments, and follow-ups).
User clicks on the "View Guideline"
System opens a page showing:
Guideline Overview: Brief information about the guideline.
Triggering Patient Result: Explanation of which patient results have triggered this order.
Actions: Recommendations on what can be done next, including a button to place an order (e.g., "Order IV Albumin Now").
User clicks the "Order Now"
System processes the order and Redirects back to Cerner to order.
COMPLETED
User Clicks on "View Results"
Redirects back to Cerner to view the completed results
OVERDUE
Due Dates can be seen
Order Execution can be done
Pending Tasks can be shared between healthcare providers for coordinated care
"Order"
System processes the order and Redirects back to Cerner to order.
"Share tasks"
Share task lists among Providers (MD), Nurses (RN), Medical Assistants (MA), and Patients.
UPCOMING
Upcoming Dates can be seen
Pending Tasks can be shared
"Share tasks"
Share task lists among Providers (MD), Nurses (RN), Medical Assistants (MA), and Patients.
"Order"
System processes the order and Redirects back to Cerner to order.
User clicks on "PATIENT REPORTED OUTCOMES MEASURE(29) SCORING"
Symptom Visualization: The app displays line graphs to track patient-reported outcomes (e.g., Physical Function, Anxiety, Depression, etc.) over time, using color-coded trends for severity levels. Users can select specific symptoms from the left-hand menu and adjust the date range to explore symptom trends.
User clicks on "PROGNOSIS SCORE"
PROGNOSIS PAGE
Displays scoring criteria, total score, and interpretation. Shows the score trend over time. Filters data by date. User can view and interact with test data.
Interactive Charts: Provide interactive charts for MELD SCORE (MODEL FOR END-STAGE LIVER DISEASE), CHILD-TURCOTTE-PUGH (CTP) SCORE, FIBROSIS-4 INDEX (FIB-4), APRI (AST TO PLATELET RATIO INDEX), LIVER STIFFNESS and NAFLD, VOCAL-PENN Score with annotations for significant changes and critical values.
Color-Coded Alerts: Highlight critical values or significant changes in trends with color-coded alerts for quick identification.

Navigation

Support center

Policies and Procedures

Archita P. Desai , MD

Division of Gastroenterology and Transplant Hepatology

Logout

SMARTLiver

Help Profile

EXISTING PATIENTS (25) REFERRED PATIENTS (15)

Search:

Name	Age	MRN	Visit Reason	Recent Visit	Labs	Patient Communication
Jesscia Taylor	47 years	64783283	Fatty Liver	12/31/2024	Incomplete	Send Notification
Ruben Hartt	28 years	83862749	NASH/ Liver Fibrosis	11/22/2024	Incomplete	Send Notification
Diana Bryan	66 years	23663200	Fatty Liver	09/10/2024	Incomplete	Send Notification
Farah Khan	54 years	54863293	Cirrhosis	12/18/2024	Completed	Send Notification
April Mayer	21 years	21863277	Fatty Liver	06/27/2024	Incomplete	Send Notification
Shawn Arnold	51 years	51863299	NASH/ Liver Fibrosis	11/07/2024	Incomplete	Send Notification
Maria hayes	73 years	73863244	Fatty Liver	12/20/2024	Incomplete	Send Notification
Linda Mike	83 years	83865457	Cirrhosis	03/30/2024	Completed	Send Notification
Ross Dann	67 years	67863283	Fatty Liver	11/22/2024	Incomplete	Send Notification
Micheal Carmy	34 years	34643747	NASH/ Liver Fibrosis	09/10/2024	Incomplete	Send Notification
Sharmitha Anand	45 years	45647569	Fatty Liver	12/18/2024	Incomplete	Send Notification
Maria hayes	24 years	24634754	Fatty Liver	12/20/2024	Completed	Send Notification

Showing 1 to 10 of 25 entries

‹‹ ‹ 1 2 › ››

SMARTLiver

Help Profile

EXISTING PATIENTS (25) REFERRED PATIENTS (15)

Search:

Name	Age	MRN	Visit Reason	Hospital Referred From	Appointment	Patient Communication
Olivia Grant	28 years	12984756	Fatty Liver	Sidney & Lois Eskenazi Hospital	12/31/2024	View
Ethan Brooks	65 years	98346277	NASH/ Liver Fibrosis	Mayo Clinic - Rochester	11/22/2024	View
Natalie Singh	53 years	73628451	Fatty Liver	Riley Hospital	09/10/2024	View
Jacob Rivera	19 years	51263748	Cirrhosis	Sidney & Lois Eskenazi Hospital	12/18/2024	View
Priya Desai	49 years	19823467	Fatty Liver	Riley Hospital	06/27/2024	View
Lucas Chen	71 years	30728463	NASH/ Liver Fibrosis	Sidney & Lois Eskenazi Hospital	11/07/2024	View
Sophia Martinez	81 years	62837415	Fatty Liver	Mayo Clinic - Rochester	12/20/2024	View
Daniel Cooper	65 years	47382910	Cirrhosis	Sidney & Lois Eskenazi Hospital	03/30/2024	View
Aisha Patel	32 years	58294736	Fatty Liver	Sidney & Lois Eskenazi Hospital	11/22/2024	View
Noah Johnson	43 years	31984725	NASH/ Liver Fibrosis	Riley Hospital	09/10/2024	View
Emma Scott	22 years	72019485	Fatty Liver	Mayo Clinic - Rochester	12/18/2024	View
Ryan Blake	38 years	24028375	Fatty Liver	Riley Hospital	12/20/2024	View

Showing 1 to 10 of 15 entries

‹‹ ‹ 1 2 › ››

SMART Liver

Name: Jessica Taylor MRN: 65374683
Sex : Female Age: 45

LAB-BASED TASKS | CLINICAL PRACTICE GUIDELINES RECOMMENDATIONS | PATIENT REPORTED OUTCOME MEASURES (29) SCORE | PROGNOSIS SCORES

Educational Materials

Send

Select materials to send to patients.

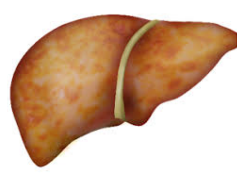

ESLD Management

Learn about managing ESLD effectively

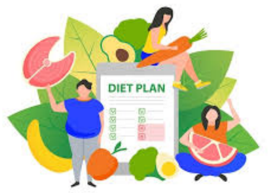

Dietary Advice

Importance of diet in ESLD management

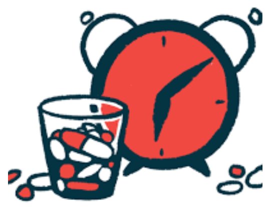

Medication Adherence

Tips for sticking to medication plans

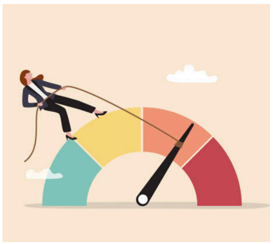

Symptom Management

Techniques and strategies for managing symptoms.

Patient Engagement

Select materials to send reminders to patients.

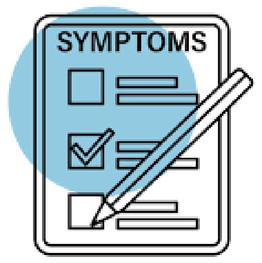

Symptom Reports

Track and report symptoms regularly

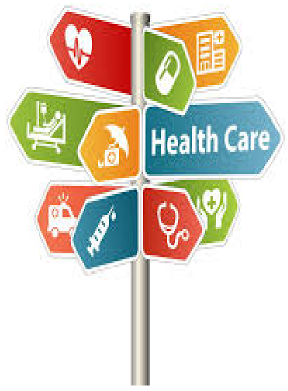

Care Plans

Please follow providers Instructions

Send

Share Tasks

Share task lists with patients, doctors, nurses, and medical assistants.

ALL(14) OVERDUE(9) UPCOMING(5)

Task Name	Due Date	Status	Action	Select
INR for coagulation	06/27/2024	Overdue	Remind	■
Paracentesis procedure	02/11/2024	Overdue	Remind	■
Ultrasound for liver fibrosis	05/21/2024	Upcoming	Remind	□
Albumin levels	12/18/2024	Upcoming	Remind	□
Liver function tests	06/27/2024	Upcoming	Remind	■
Albumin levels	05/07/2024	Overdue	Remind	□
Hepatitis B vaccine	11/27/2024	Overdue	Remind	□

Choose Recipients

SMART Liver

Name: Jessica Taylor MRN: 65374683
Sex : Female Age: 45

LAB-BASED TASKS | CLINICAL PRACTICE GUIDELINES RECOMMENDATIONS | PATIENT REPORTED OUTCOME MEASURES (29) SCORE | PROGNOSIS SCORES

Educational Materials

Send

Managing ESLD Complications

Ascites	■
Hepatic Encephalopathy	□
Varices	□
Pleural Effusion	■
Hepatocellular Carcinoma	■

Procedures and Treatments

Ablation Therapy	■
Embolization	□
Lab Tests	■
Liver Biopsy	■
Liver Transplant	□

Healthy Living Resources

Advance Care Planning	■
Alcohol	□
Cannabis and Smoking	□
Care Partner Support	■
Exercise	□

Symptom Relief and Management

Anxiety	■
Depression	■
Itching	□
Muscle Cramps	■
Pain	□

Patient Engagement

Symptom Reporting Reminder

General Symptoms
Fatigue, Skin Changes, Weight Fluctuations, Abdominal Pain

Gastrointestinal Symptoms
Bowel Habits, Nausea and Vomiting, Color of Stool, Gas or Bloating

Urinary Symptoms
Urine Color, Peeing Habits

Other Symptoms
Type ....

Care Plan

Please follow providers instructions

Send

Share Tasks

ALL(14) OVERDUE(9) UPCOMING(5)

Task Name	Due Date	Status	Select
INR for coagulation	06/27/2024	Overdue	■
Paracentesis procedure	02/11/2024	Overdue	□
Ultrasound for liver fibrosis	05/21/2024	Overdue	■
Albumin levels	12/18/2024	Upcoming	□
Liver function tests	06/27/2024	Upcoming	□
Albumin levels	05/07/2024	Overdue	■

Send Message

Type.........

Choose Recipients

Recipient	Select
Patient	■
Providers (MD)	□
Medical Assistants (MA)	■
Nurses (RN)	□

Send

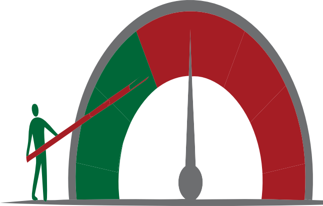

SMART Liver

Name: Jessica Taylor MRN: 65374683
Sex : Female Age: 45

LAB-BASED TASKS | CLINICAL PRACTICE GUIDELINES RECOMMENDATIONS | PATIENT REPORTED OUTCOME MEASURES (29) SCORE | PROGNOSIS SCORES

- ALL(15)
- CONDITION/STATUS(2)
- ORDER SETS(5)
- PATIENT CARE(0)
- NUTRITION SERVICES(2)
- CONTINUOUS INFUSIONS(1)
- LABORATORY SERVICES(2)
- RADIOLOGY SERVICES(2)
- CARDIOVASCULAR SERVICES(2)
- PULMONARY SERVICES(2)
- SURGICAL PROCEDURES(2)
- OTHER DEPARTMENTS(2)
- MEDICAL SUPPLIES(2)
- NON CATEGORIZED(2)

ALL(15) REQUIRED(5) NOT REQUIRED(5) REMIND ME LATER(5)

Search:

Task Name	Guideline	Necessity	Action
Administer IV Albumin	View	Remind Me Later	Order Now
INR for coagulation	View	Required	Order Now
Hepatitis B vaccine	View	Required	Order Now
Paracentesis procedure	View	Not Required	Order Now
ALT and AST levels	View	Remind Me Later	Order Now
INR for coagulation	View	Not Required	Order Now
Pneumococcal vaccine	View	Remind Me Later	Order Now

Showing 1 to 10 of 15 entries

« ‹ 1 2 › »

SMART Liver

Name: Jessica Taylor **MRN:** 65374683
Sex : Female **Age:** 45

LAB-BASED TASKS | CLINICAL PRACTICE GUIDELINES RECOMMENDATIONS | PATIENT REPORTED OUTCOME MEASURES (29) SCORE | PROGNOSIS SCORES

Process Based Measure

Large-Volume Paracentesis: 6.2 liters

Task Information

Provide IV Albumin for Paracentesis > 5L

The patient should be given IV albumin (6-8 g/L removed) after a paracentesis exceeding 5 liters.

Clinical Practice Guideline Details

Guideline Content

Administering IV albumin post-large-volume paracentesis to prevent circulatory dysfunction.

Patient Results Triggering the CPG

Procedure	*Volume Removed*	*Triggering Condition*
Paracentesis > 5L performed on 6/14/2024	6.2 litres	Paracentesis volume exceeded 5 liters, indicating the need for IV albumin administration.

SMART Liver

Name: Jessica Taylor **MRN:** 65374683
Sex : Female **Age:** 45

LAB-BASED TASKS | CLINICAL PRACTICE GUIDELINES RECOMMENDATIONS | PATIENT REPORTED OUTCOME MEASURES (29) SCORE | PROGNOSIS SCORES

- ALL(15)
- CONDITION/STATUS(2)
- ORDER SETS(5)
- PATIENT CARE(0)
- NUTRITION SERVICES(2)
- CONTINUOUS INFUSIONS(1)
- LABORATORY SERVICES(2)
- RADIOLOGY SERVICES(2)
- CARDIOVASCULAR SERVICES(2)
- PULMONARY SERVICES(2)
- SURGICAL PROCEDURES(2)
- OTHER DEPARTMENTS(2)
- MEDICAL SUPPLIES(2)
- NON CATEGORIZED(2)

LABS IN IU HEALTH (5) | LABS OUTSIDE IU HEALTH (5)

Search:

Task Name	Due Date	Status	Action	Patient Communication
Hemogram-Platelets-WBC	08/20/2024	Upcoming	Order Now	Send Notification
Ultrasound	08/12/2024	Completed	View Results	Send Notification
Vital Signs	08/14/2024	Completed	View Results	Send Notification
Gastroenterology Procedures	08/18/2024	Overdue	Order Now	Send Notification
INR for coagulation	08/20/2024	Upcoming	Order Now	Send Notification

Showing 1 to 5 of 5 entries

« ‹ 1 › »

SMART Liver

Name: Jessica Taylor **MRN:** 65374683
Sex : Female **Age:** 45

LAB-BASED TASKS | CLINICAL PRACTICE GUIDELINES RECOMMENDATIONS | PATIENT REPORTED OUTCOME MEASURES (29) SCORE | PROGNOSIS SCORES

- ALL(15)
- CONDITION/STATUS(2)
- ORDER SETS(5)
- PATIENT CARE(0)
- NUTRITION SERVICES(2)
- CONTINUOUS INFUSIONS(1)
- LABORATORY SERVICES(2)
- RADIOLOGY SERVICES(2)
- CARDIOVASCULAR SERVICES(2)
- PULMONARY SERVICES(2)
- SURGICAL PROCEDURES(2)
- OTHER DEPARTMENTS(2)
- MEDICAL SUPPLIES(2)
- NON CATEGORIZED(2)

LABS IN IU HEALTH (5) | LABS OUTSIDE IU HEALTH (5)

Search:

Task Name	Due Date	Status	Action	Patient Communication
Liver Function Panel	08/20/2024	Upcoming	Order Now	Send Notification
Abdominal CT Scan	08/12/2024	Completed	View Results	Send Notification
ECG/EKG Test	08/14/2024	Completed	View Results	Send Notification
Discharge Planning Evaluation	08/18/2024	Overdue	Order Now	Send Notification
Fluid Balance Monitoring	08/20/2024	Upcoming	Order Now	Send Notification

Showing 1 to 5 of 5 entries

« ‹ 1 › »

SMART Liver

Name: Jessica Taylor **MRN:** 65374683
Sex : Female **Age:** 45

LAB-BASED TASKS | CLINICAL PRACTICE GUIDELINES RECOMMENDATIONS | PATIENT REPORTED OUTCOME MEASURES (29) SCORE | PROGNOSIS SCORES

MELD SCORE 3.0 (MODEL FOR END-STAGE LIVER DISEASE)

CTP (CHILD-TURCOTTE-PUGH) SCORE

FIB-4 (FIBROSIS-4 INDEX)

APRI (AST TO PLATELET RATIO INDEX)

LIVER STIFFNESS MEASUREMENT

NAFLD (NON-ALCOHOLIC FATTY LIVER DISEASE) FIBROSIS SCORE

VOCAL-PENN CIRRHOSIS SURGICAL RISK SCORE

MELD 3.0 (Model For End-Stage Liver Disease) Score

View Normal Ranges

Date Range

From mm/dd/yyyy To mm/dd/yyyy Done

Patient's Clinical Values for MELD-Na Score Calculation

Date 9/17/2024

Parameter	Value
Creatinine	1.8 mg/dL
Bilirubin	2.5 mg/dL
INR	1.6
Sodium	130 mEq/L
Dialysis status	No
Age at Listing	<18 years

Results
MELD-Na Score: 25.23

Mortality Rate: 19.6%

Interpretation: With a MELD-Na score of 25, this patient falls into the 20-29 range, indicating a high risk of mortality and significant liver impairment. This patient may need closer monitoring and possibly evaluation for liver transplantation.

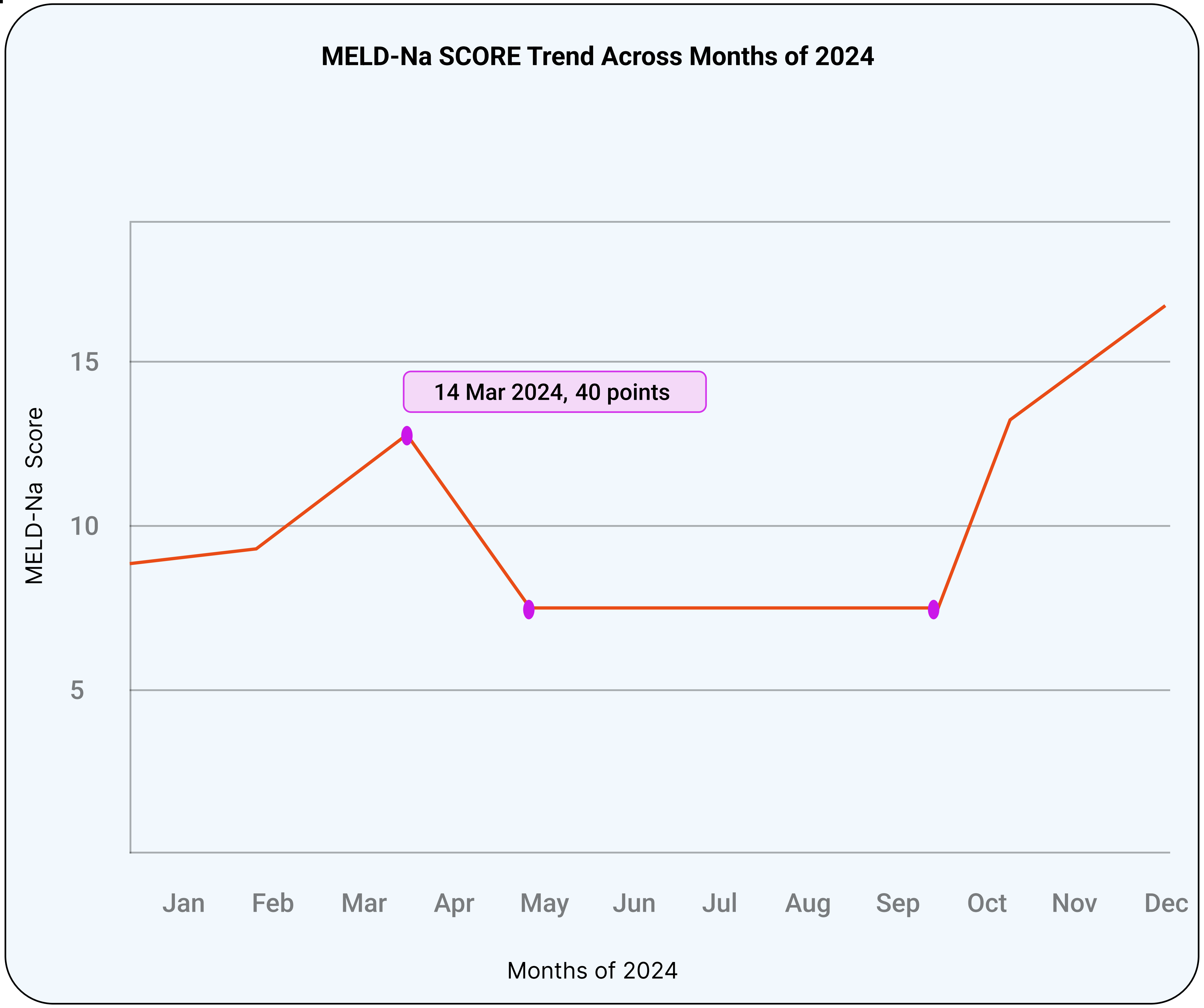

SMART Liver

Name: Jessica Taylor **MRN:** 65374683
Sex : Female **Age:** 45

LAB-BASED TASKS | CLINICAL PRACTICE GUIDELINES RECOMMENDATIONS | PATIENT REPORTED OUTCOME MEASURES (29) SCORE | PROGNOSIS SCORES

- MELD SCORE 3.0 (MODEL FOR END-STAGE LIVER DISEASE)
- CTP (CHILD-TURCOTTE-PUGH) SCORE
- FIB-4 (FIBROSIS-4 INDEX)
- APRI (AST TO PLATELET RATIO INDEX)
- LIVER STIFFNESS MEASUREMENT
- NAFLD (NON-ALCOHOLIC FATTY LIVER DISEASE) FIBROSIS SCORE
- VOCAL-PENN CIRRHOSIS SURGICAL RISK SCORE

MELD 3.0 (Model For End-Stage Liver Disease) Score

Parameter	Normal Range	Notes
Creatinine	0.7 - 1.3 mg/dL	62 - 115 μmol/L
Bilirubin	0.3 - 1.9 mg/dL	5.13 - 32.49 μmol/L
INR	0.8 - 1.2	N/A
Sodium	0.7 - 1.3 mEq/L	136 - 145 mmol/L
	3.5 - 5.5 g/dL	35 - 55 g/L

MELD-Na Score	Class Interpretation
40 or more	Extremely high risk; decompensated liver; immediate need.
30-39	Very high risk; severe liver disease; urgent evaluation.
20-29	High risk; significant liver impairment.
10-19	Moderate risk; early liver dysfunction.
<9	Low risk; well-compensated liver disease.

Date Range

From mm/dd/yyyy **To** mm/dd/yyyy Done

Hide Normal Ranges

Patient's Clinical Values for MELD-Na Score Calculation

Date 9/17/2024

Parameter	Value
Creatinine	1.8 mg/dL
Bilirubin	2.5 mg/dL
INR	1.6
Sodium	130 mEq/L
Dialysis status	No
Age at Listing	

Results: MELD-Na Score: 25.23, **Mortality Rate:** 19.6%

Interpretation: With a MELD-Na score of 25, this patient falls into the 20-29 range, indicating a high risk of mortality and significant liver impairment. This patient may need closer monitoring and possibly evaluation for liver transplantation.

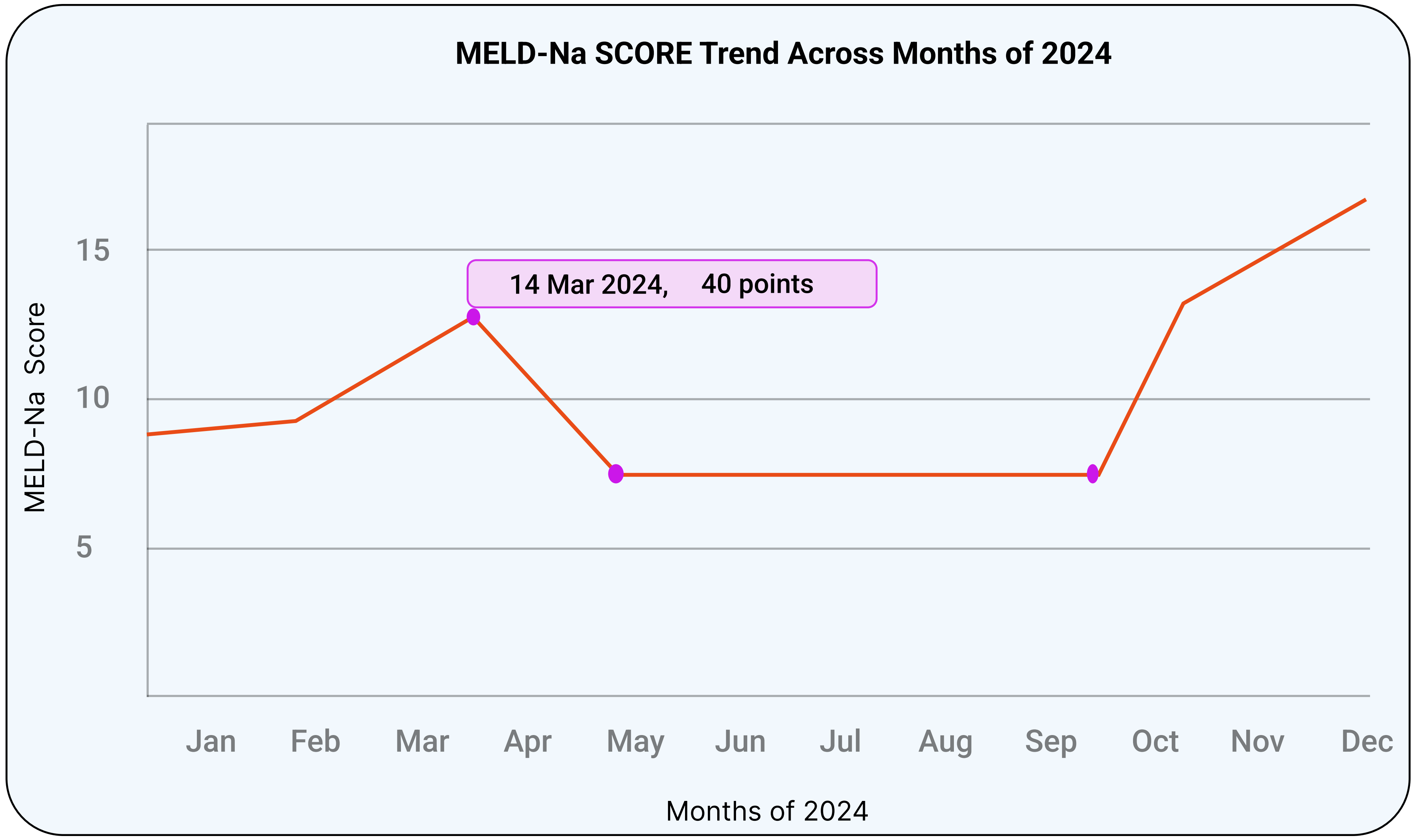

SMART Liver

Name: Jessica Taylor **MRN:** 65374683
Sex : Female **Age:** 45

LAB-BASED TASKS | CLINICAL PRACTICE GUIDELINES RECOMMENDATIONS | PATIENT REPORTED OUTCOME MEASURES (29) SCORE | PROGNOSIS SCORES

MELD SCORE (MODEL FOR END-STAGE LIVER DISEASE)

CTP (CHILD-TURCOTTE-PUGH) SCORE

FIBROSIS-4 INDEX (FIB-4)

APRI (AST TO PLATELET RATIO INDEX)

LIVER STIFFNESS MEASUREMENT

NAFLD (NON-ALCOHOLIC FATTY LIVER DISEASE) FIBROSIS SCORE

VOCAL-PENN CIRRHOSIS SURGICAL RISK SCORE

Child-Turcotte-Pugh (CTP) Score

Criterion	1 point		2 points		3 points	
Encephalopathy	None		Grade I - II		Grade III - IV	
Ascites	None		Moderate		Tense	
Total Bilirubin	<2 mg/dL	<34.2 umol/L	2-3 mg/dL	34.2-51.3 umol/L	>3 mg/dL	>51.3 umol/L
Albumin	>3.5 g/dL	>35 g/L	2.8-3.5 g/dL	28-35 g/L	<2.8 g/dL	<28 g/L
INR	<1.7		1.7 - 2.3		> 2.3	

CTP score is obtained by adding the score for each parameter.

CTP class	Points	Class Interpretation
A	5-6	Well-compensated liver disease.
B	7-9	Significant functional compromise.
C	10-15	Decompensated liver disease with poor.

Date Range

From mm/dd/yyyy To mm/dd/yyyy Done

Patient's Clinical Values for CTP Score Calculation

Date 9/17/2024

Parameter	Value	Points
Encephalopathy	None	1 point
Ascites	Moderate	2 points
Total Bilirubin	2.5 mg/dL	2 points
Albumin	3.0 g/dL	2 points
INR	1.8	2 points

Results

Total CTP Score: 9 points

CTP Class: B (Significant functional compromise)

Interpretation: The patient falls into CTP Class C, indicating decompensated liver disease with poor prognosis. This class suggests severe cirrhosis with high mortality risk, and the patient may require urgent liver transplantation consideration.

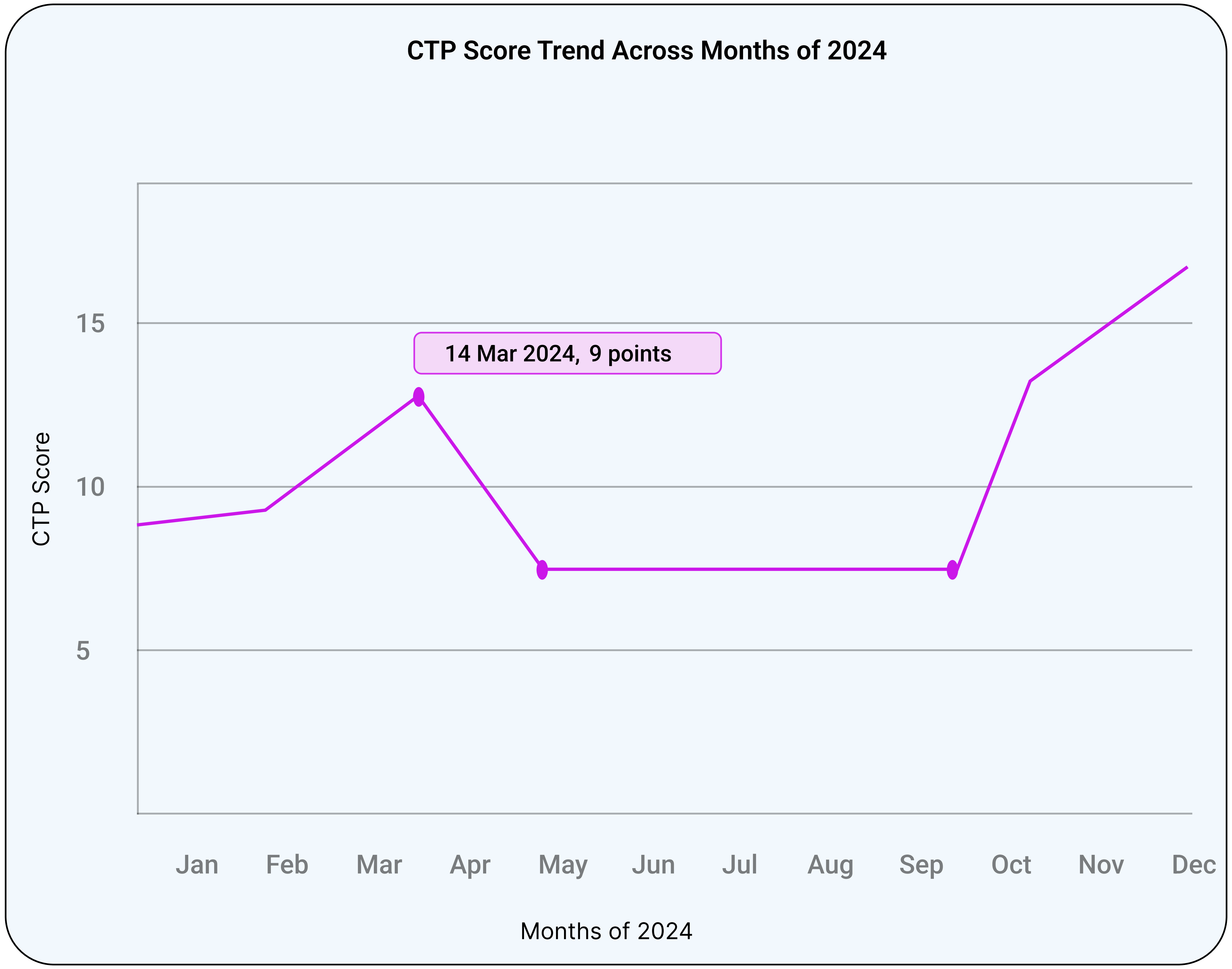

SMART Liver

Name: Jessica Taylor **MRN:** 65374683
Sex : Female **Age:** 45

LAB-BASED TASKS | CLINICAL PRACTICE GUIDELINES RECOMMENDATIONS | PATIENT REPORTED OUTCOME MEASURES (29) SCORE | PROGNOSIS SCORES

- MELD SCORE (MODEL FOR END-STAGE LIVER DISEASE)
- CTP (CHILD-TURCOTTE-PUGH) SCORE
- FIBROSIS-4 INDEX (FIB-4)
- APRI (AST TO PLATELET RATIO INDEX)
- LIVER STIFFNESS MEASUREMENT
- NAFLD (NON-ALCOHOLIC FATTY LIVER DISEASE) FIBROSIS SCORE
- VOCAL-PENN CIRRHOSIS SURGICAL RISK SCORE

Fibrosis-4 (FIB4) Index for Liver Fibrosis

Parameter	Normal Range
Age	years
AST (Aspartate Aminotransferase)	15 - 41 U/L
ALT (Alanine Aminotransferase)	1 - 35 U/L
Platelet count	150 - 350 × $10^3/\mu L$

FIB-4 Score	Interpretation
< 1.30	Low likelihood of significant fibrosis (F0-F1); Normal.
1.30 - 2.67	Intermediate risk of fibrosis (F2);Further evaluation recommended.
> 2.67	High likelihood of advanced fibrosis (F3-F4); Abnormal.

Date Range

From mm/dd/yyyy To mm/dd/yyyy Done

Patient's Clinical Values for FIB-4 Score Calculation

Date 9/17/2024

Parameter	Value
Age	55 years
AST (Aspartate Aminotransferase)	80 U/L
ALT (Alanine Aminotransferase)	65 U/L
Platelet count	150 × $10^3/\mu L$

Results

FIB-4 Score: 3.64

Interpretation: With a FIB-4 score of 3.64, this patient falls into the > 3.25 range, suggesting a high likelihood of advanced liver fibrosis (F3-F4). This is considered abnormal, and further diagnostic workup or specialist referral is typically recommended.

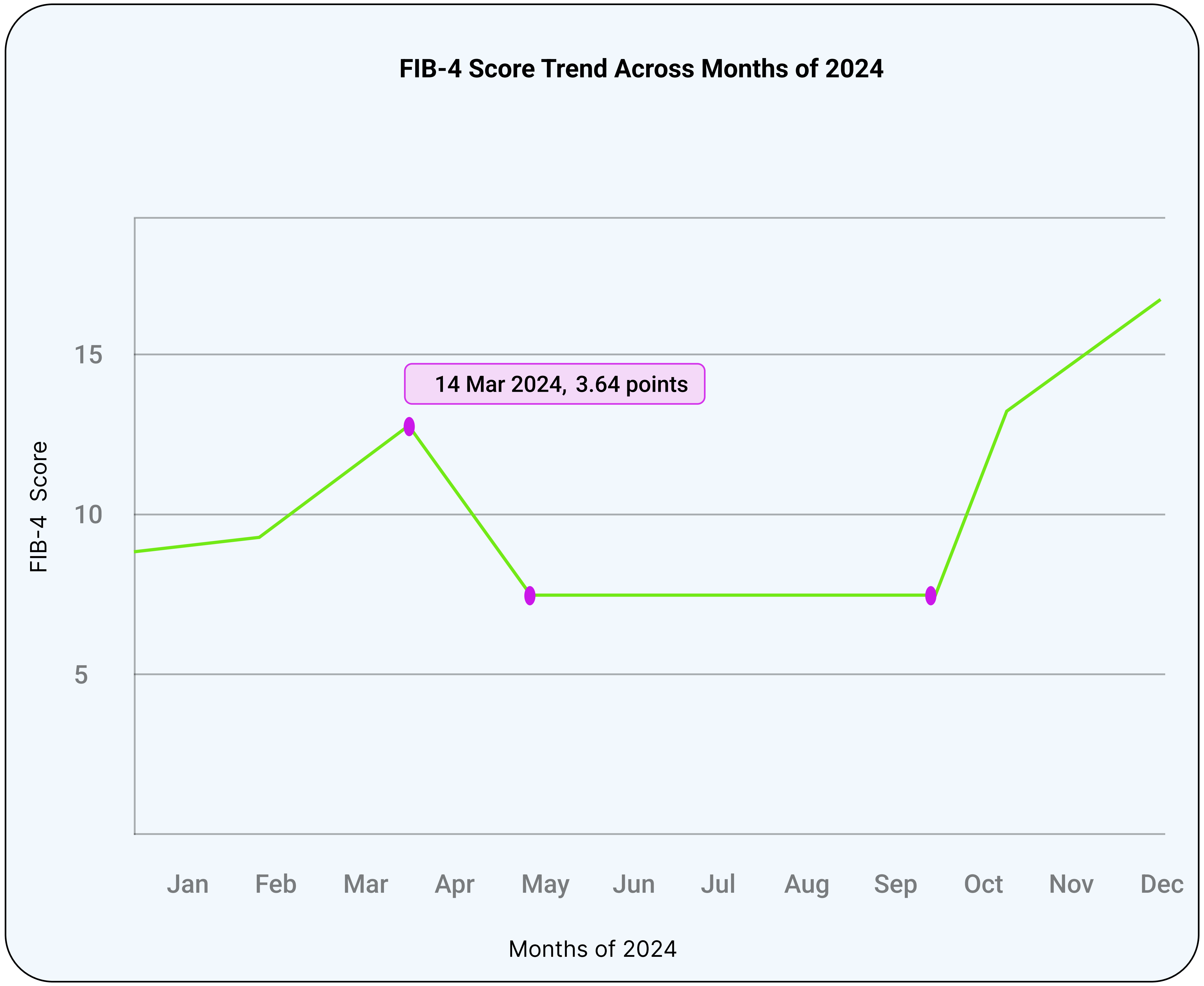

SMART Liver

Name: Jessica Taylor **MRN:** 65374683
Sex : Female **Age:** 45

LAB-BASED TASKS | CLINICAL PRACTICE GUIDELINES RECOMMENDATIONS | PATIENT REPORTED OUTCOME MEASURES (29) SCORE | PROGNOSIS SCORES

- MELD SCORE (MODEL FOR END-STAGE LIVER DISEASE)
- CTP (CHILD-TURCOTTE-PUGH) SCORE
- FIB-4 (FIBROSIS-4 INDEX)
- APRI (AST TO PLATELET RATIO INDEX)
- LIVER STIFFNESS
- NAFLD (NON-ALCOHOLIC FATTY LIVER DISEASE) FIBROSIS SCORE
- VOCAL-PENN CIRRHOSIS SURGICAL RISK SCORE

AST to Platelet Ratio Index (APRI)

Parameter	Normal Range
AST (Aspartate Aminotransferase)	15 - 41 U/L
AST Upper Limit of Normal (ULN)	40 U/L (standard value used in APRI calculations)
Platelet Count	150 - 350 × $10^3/\mu L$

APRI Score	Interpretation
< 0.5	Low likelihood of significant fibrosis; normal or minimal fibrosis (F0-F1).
0.5 - 1.5	Possible mild to moderate fibrosis (F2); further investigation may be needed.
> 1.5	High likelihood of significant fibrosis or cirrhosis (F3-F4); abnormal.

Date Range

From mm/dd/yyyy To mm/dd/yyyy Done

Patient's Clinical Values for APRI Score Calculation

Date 9/17/2024

Parameter	Value
AST level	60 U/L
AST Upper Limit of Normal (ULN)	40 U/L
Platelet count	120 × 10^9/L

Results
APRI Score: 1.25

Interpretation: With an APRI score of 1.25, the patient falls in the 0.5 - 1.5 range, indicating a possible mild to moderate fibrosis. Further investigation, such as elastography or liver biopsy, may be needed to confirm the extent of liver fibrosis.

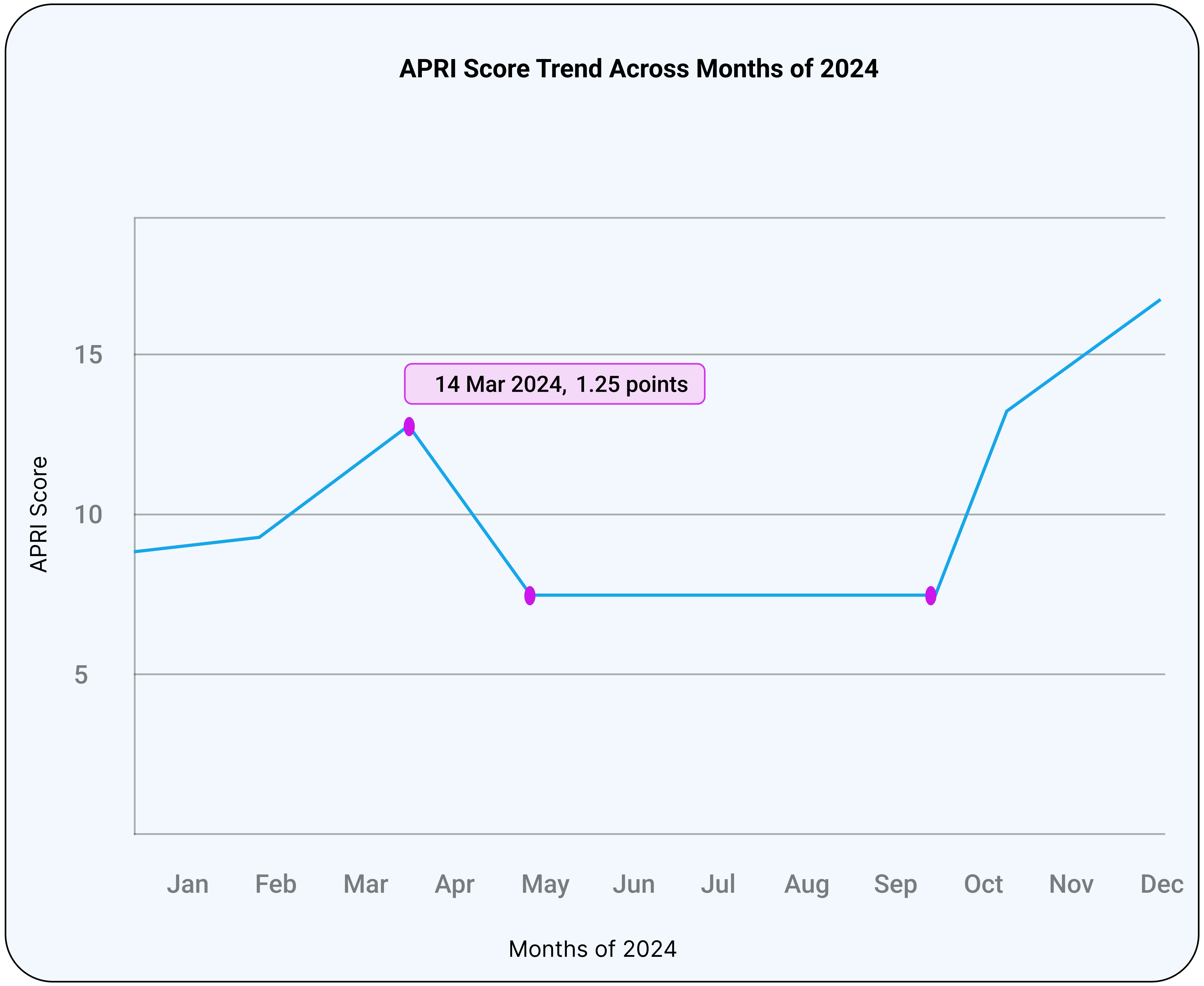

SMART Liver

Name: Jessica Taylor **MRN:** 65374683
Sex : Female **Age:** 45

LAB-BASED TASKS | CLINICAL PRACTICE GUIDELINES RECOMMENDATIONS | PATIENT REPORTED OUTCOME MEASURES (29) SCORE | PROGNOSIS SCORES

- MELD SCORE (MODEL FOR END-STAGE LIVER DISEASE)
- CTP (CHILD-TURCOTTE-PUGH) SCORE
- FIB-4 (FIBROSIS-4 INDEX)
- APRI (AST TO PLATELET RATIO INDEX)
- LIVER STIFFNESS MEASUREMENT
- NAFLD (NON-ALCOHOLIC FATTY LIVER DISEASE) FIBROSIS SCORE
- VOCAL-PENN CIRRHOSIS SURGICAL RISK SCORE

Liver Stiffness Measurement (kPa)

Condition	F0-F1	F2	F3	F4
Hepatitis B	2-7 kPa	8-9 kPa	8-11 kPa	18 kPa or higher
Hepatitis C	2-7 kPa	8-9 kPa	9-14 kPa	14 kPa or higher
HIV/HCV Coinfection	2-7 kPa	7-11 kPa	11-14 kPa	14 kPa or higher
Cholestatic Disease	2-7 kPa	7-9 kPa	9-17 kPa	17 kPa or higher
Nonalcoholic Fatty Liver Disease (NAFLD OR NASH)	2-7 kPa	7.5-10 kPa	10-14 kPa	14 kPa or higher
Alcohol Related Liver Disease	2-7 kPa	7-11 kPa	11-19 kPa	19 kPa or higher

Stage	Class Interpretation
F0 - F1	No or Mild Liver Scarring
F2	Moderate Liver Scarring
F3	Severe Liver Scarring
F4	Cirrhosis

Date Range

From mm/dd/yyyy **To** mm/dd/yyyy Done

Patient's Liver Stiffness (kPa) Value

Date 9/17/2024

Results

**Liver Stiffness Measurement**	12 kPa
**Patient Condition**	Hepatitis
**Stage**	F3
**Class**	Severe Liver Scarring

Interpretation: Liver stiffness of 12 kPa falls within the F3 (Severe Liver Scarring) category for Hepatitis C.
This indicates the patient has severe fibrosis.

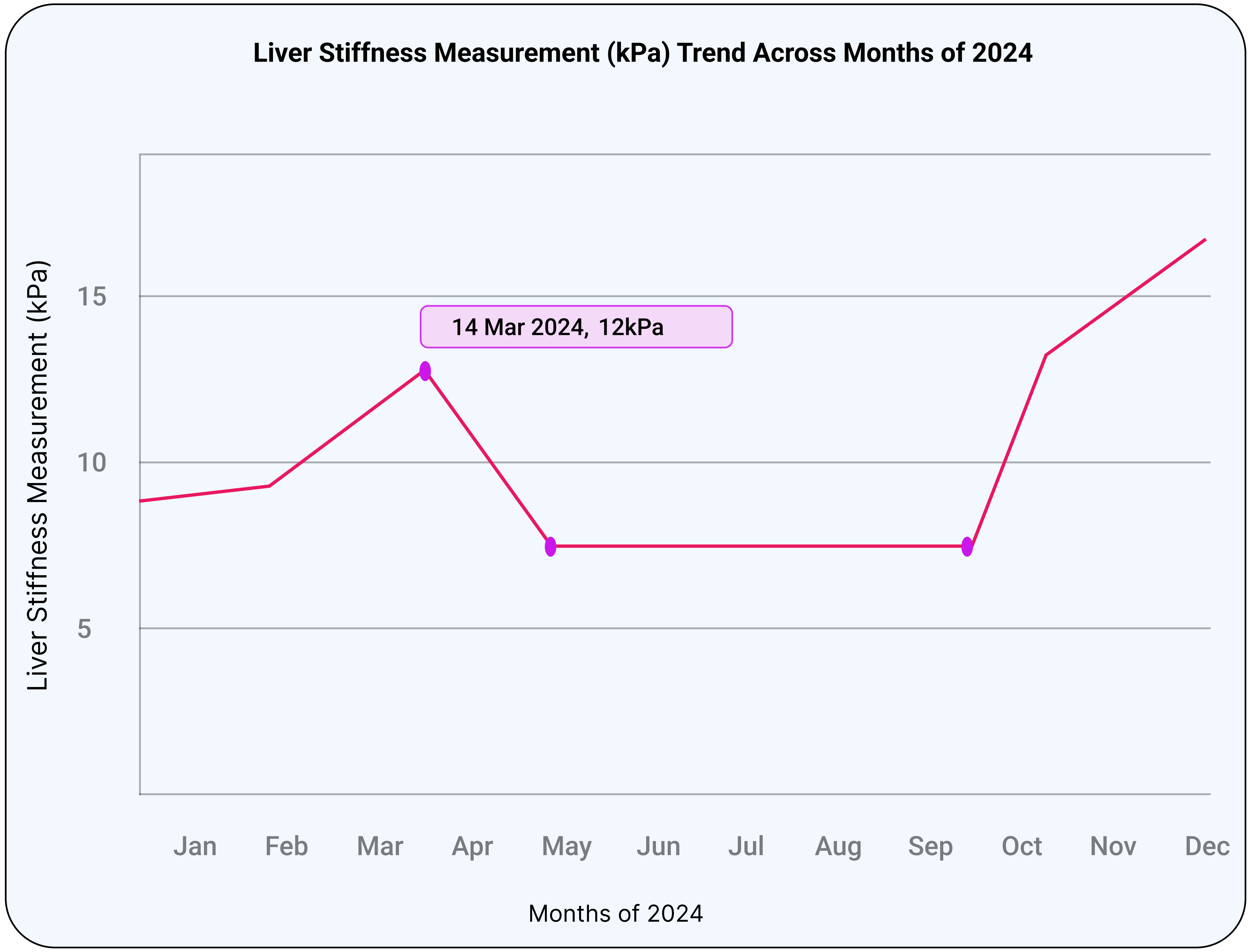

SMART Liver

Name: Jessica Taylor **MRN:** 65374683
Sex : Female **Age:** 45

LAB-BASED TASKS | CLINICAL PRACTICE GUIDELINES RECOMMENDATIONS | PATIENT REPORTED OUTCOME MEASURES (29) SCORE | PROGNOSIS SCORES

- MELD SCORE (MODEL FOR END-STAGE LIVER DISEASE)
- CTP (CHILD-TURCOTTE-PUGH) SCORE
- FIB-4 (FIBROSIS-4 INDEX)
- APRI (AST TO PLATELET RATIO INDEX)
- LIVER STIFFNESS MEASUREMENT
- NAFLD (NON-ALCOHOLIC FATTY LIVER DISEASE) FIBROSIS SCORE
- VOCAL-PENN CIRRHOSIS SURGICAL RISK SCORE

NAFLD (Non-Alcoholic Fatty Liver Disease) Fibrosis Score

Parameter	Normal Range
Age	Any age (years)
BMI (Body Mass Index)	20 - 25 kg/m²
Impaired Fasting Glucose (IFG)/Diabetes	No (0) / Yes (+1)
AST (Aspartate Aminotransferase)	15 - 41 U/L
ALT (Alanine Aminotransferase)	1 - 35 U/L
Platelet Count	150 - 350 × 10³/μL
Albumin	3.5 - 5.5 g/dL

NAFLD Fibrosis Score	Class Interpretation
<-1.455	Low risk of advanced fibrosis; No need for further immediate testing.
-1.455 to 0.676	Indeterminate; Further testing may be needed.
>0.676	High risk of advanced fibrosis; Consider further evaluation or biopsy.

Date Range

From mm/dd/yyyy To mm/dd/yyyy Done

Patient's Clinical Values for NAFLD Score Calculation

Date 9/17/2024

Parameter	Values
Age	55 years
BMI	28 kg/m²
Diabetes	Yes (+1)
AST	48 U/L
ALT	36 U/L
Platelet Count	180 × 10³/μL
Albumin	4.0 g/dL

Results

NAFLD Fibrosis Score: 0.332

Interpretation: With a NAFLD Fibrosis Score of 0.332, the patient falls in the indeterminate range (-1.455 to 0.676), suggesting that further testing, such as elastography, might be needed to better assess the degree of liver fibrosis.

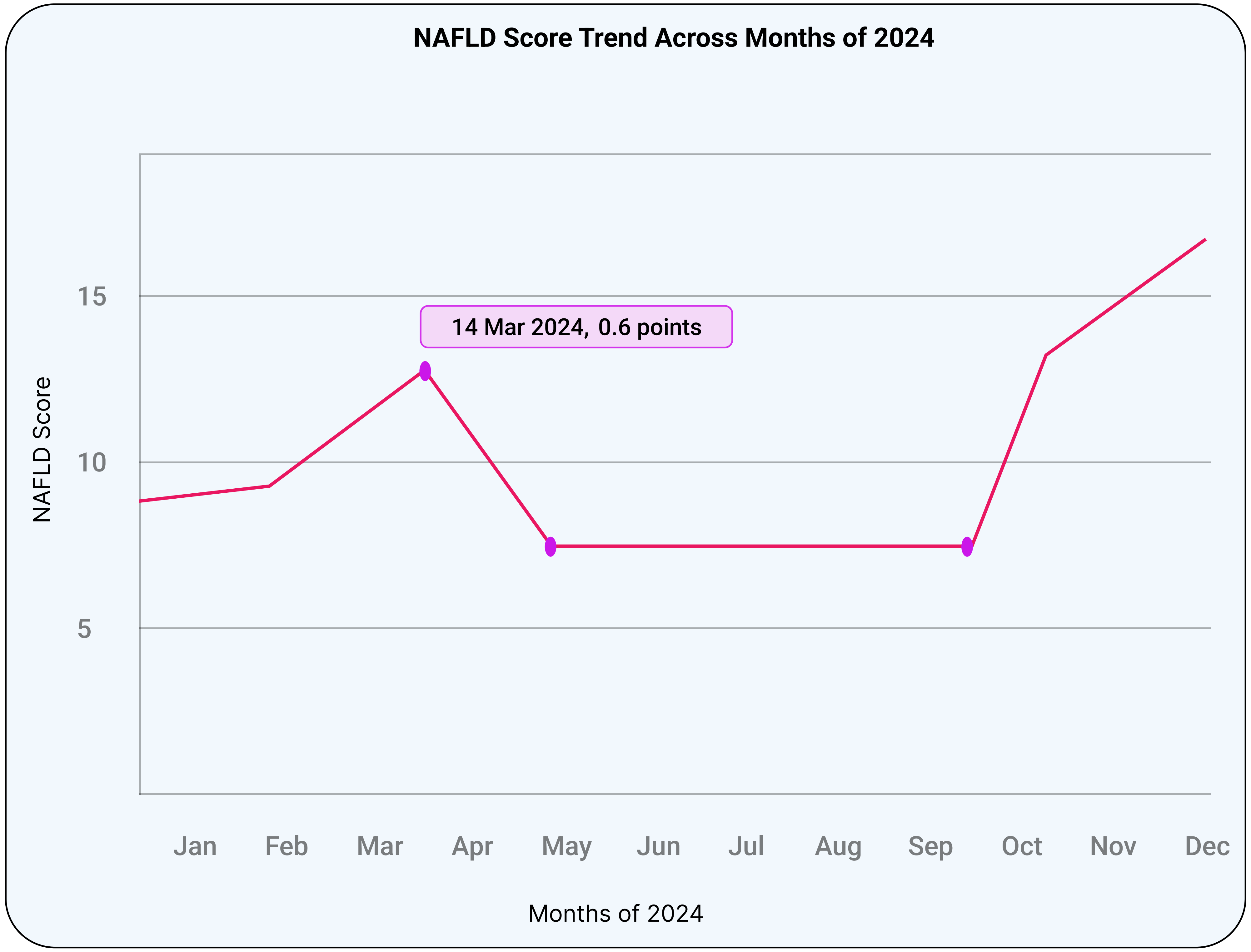

VOCAL-Penn Cirrhosis Surgical Risk Score

Date 9/17/2024

Parameter	Normal Range
Age	Any age (years)
Albumin	g/dL
Total Bilirubin	mg/dL
Platelet Count	x1,000/μL
BMI > 30	Yes/No
MASLD	Yes/No
ASA Score	2/3/4
Emergency	Yes/No
Surgery Type	Abdominal-Lap/Abdominal-Open/Abdominal Wall/Vascular/Major Orthopedic/Chest/cardiac

	98.8%
	98.8%
	98.8%
	98.8%

Interpretation: The postoperative outcomes scores indicate an extremely high risk of mortality (98.8%) within 30, 90, and 180 days after surgery, with a relatively lower but still significant risk (9.2%) of liver decompensation within 90 days. These results suggest surgery poses critical risks, likely outweighing potential benefits.

SMART Liver

Name: Jessica Taylor MRN: 65374683
Sex : Female Age: 45

LAB-BASED TASKS | CLINICAL PRACTICE GUIDELINES RECOMMENDATIONS | PATIENT REPORTED OUTCOME MEASURES (29) SCORE | PROGNOSIS SCORES

- ALL
- PHYSICAL FUNCTION
- ANXIETY
- DEPRESSION
- FATIGUE
- SLEEP DISTURBANCE
- SOCIAL PARTICIPATION
- COGNITIVE FUNCTION

PROMIS-29 (Patient-Reported Outcomes Measurement Score)

Domain	Total Score
Physical Function	40.4
Anxiety	59.5
Depression	58.9
Fatigue	50.9
Sleep Disturbance	54.5
Social Participation	48.1
Pain Interference	55.7
Cognitive Function	34.4

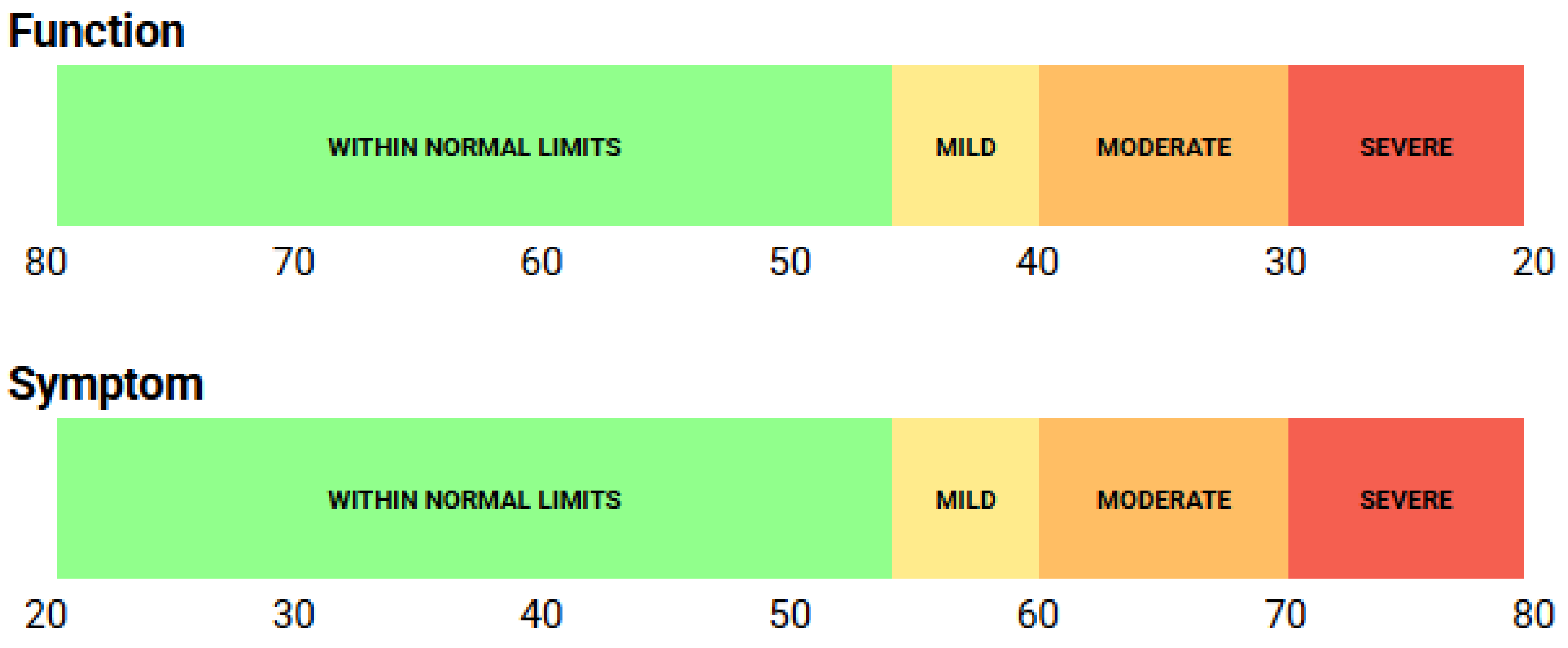

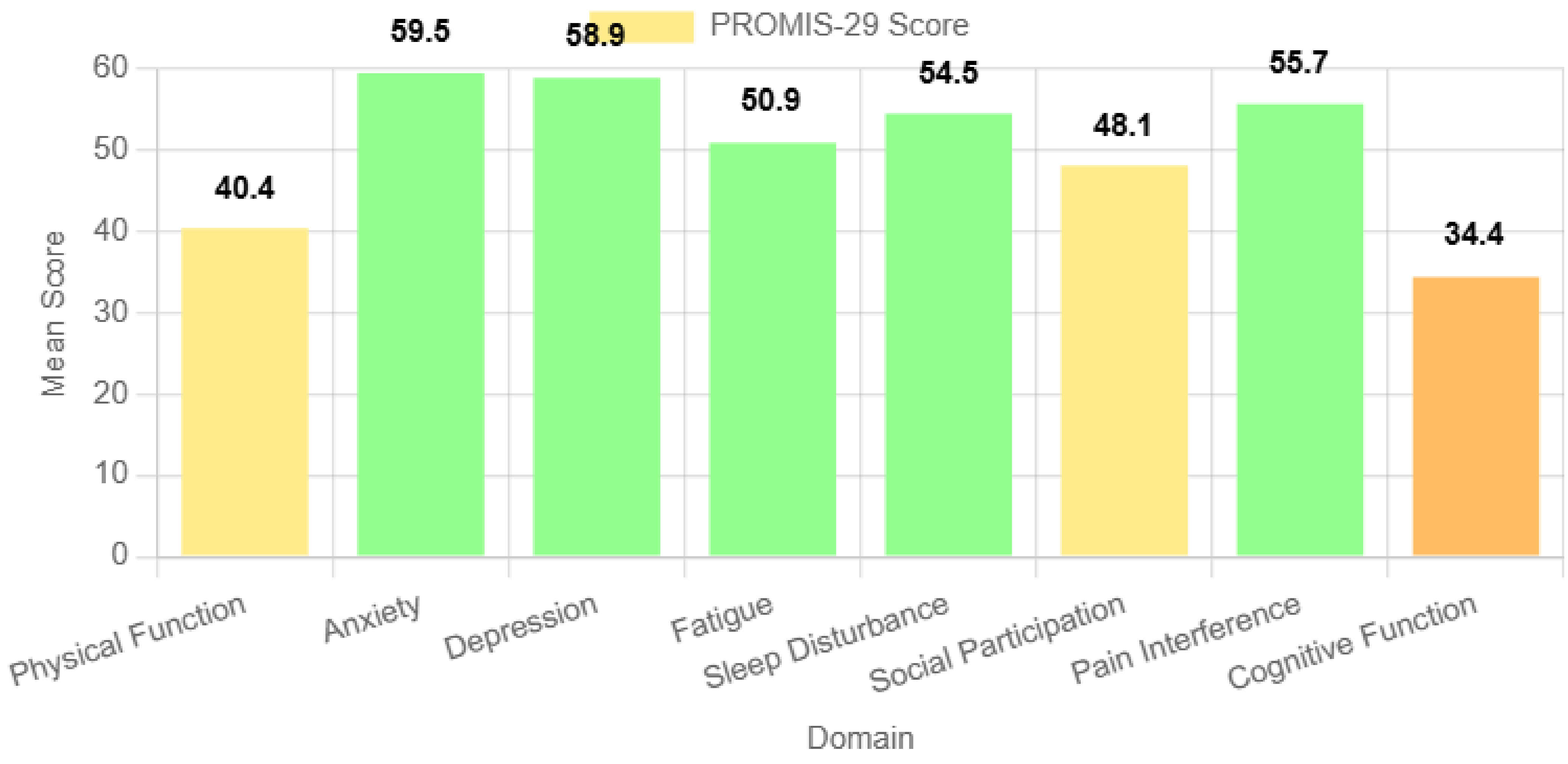

SMART Liver

Name: Jessica Taylor **MRN:** 65374683
Sex : Female **Age:** 45

LAB-BASED TASKS | CLINICAL PRACTICE GUIDELINES RECOMMENDATIONS | PATIENT REPORTED OUTCOME MEASURES (29) SCORE | PROGNOSIS SCORES

- ALL
- PHYSICAL FUNCTION
- ANXIETY
- DEPRESSION
- FATIGUE
- SLEEP DISTURBANCE
- SOCIAL PARTICIPATION
- PAIN INTERFERENCE
- COGNITIVE FUNCTION

Physical Function - PROMIS-29 SCORE

From mm/dd/yyyy To mm/dd/yyyy Done

Function

WITHIN NORMAL LIMITS	MILD	MODERATE	SEVERE

80 70 60 50 40 30 20

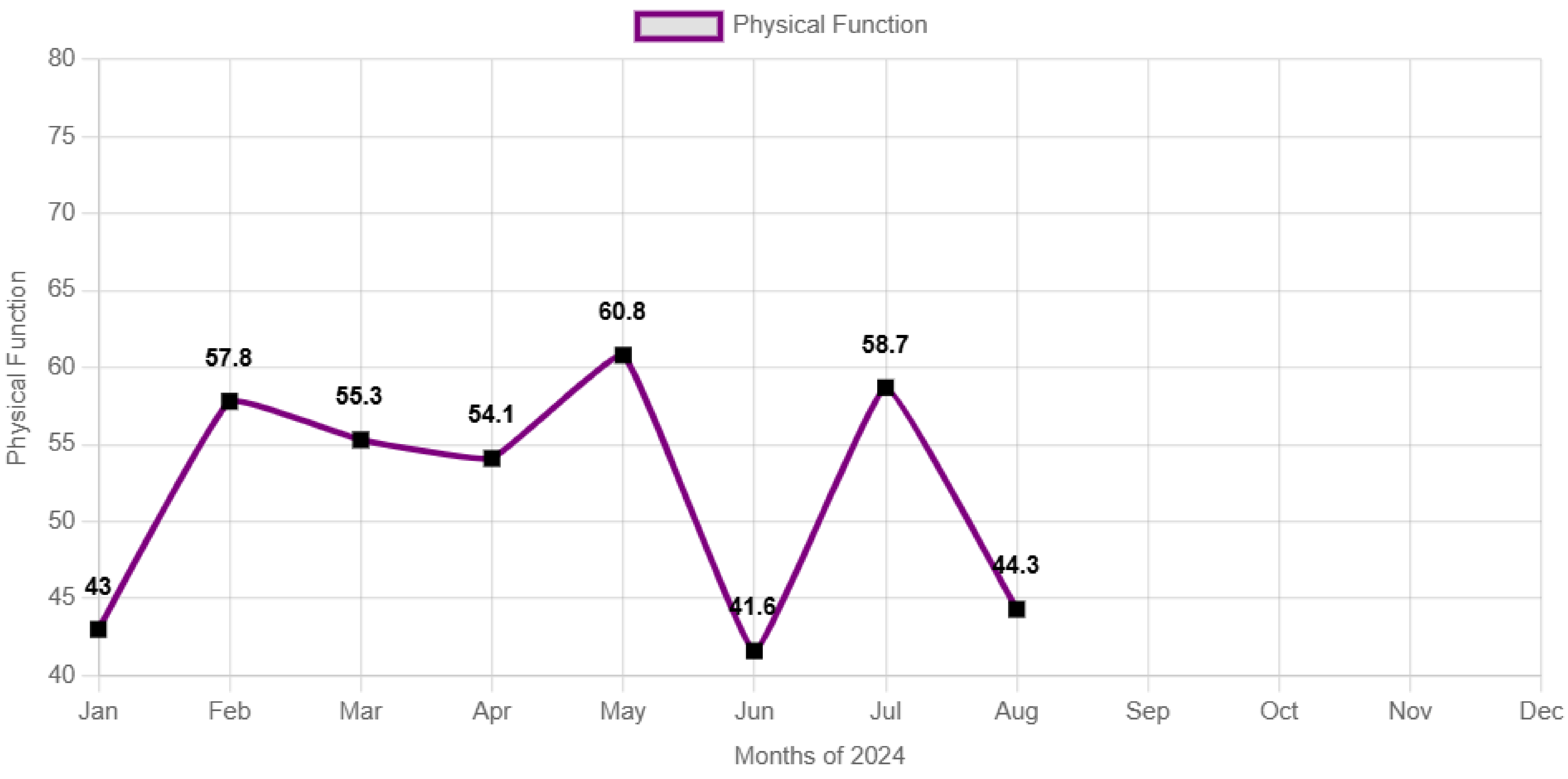

SMART Liver

Name: Jessica Taylor **MRN:** 65374683
Sex : Female **Age:** 45

LAB-BASED TASKS | CLINICAL PRACTICE GUIDELINES RECOMMENDATIONS | PATIENT REPORTED OUTCOME MEASURES (29) SCORE | PROGNOSIS SCORES

- ALL
- PHYSICAL FUNCTION
- ANXIETY
- DEPRESSION
- FATIGUE
- SLEEP DISTURBANCE
- SOCIAL PARTICIPATION
- PAIN INTERFERENCE
- PAIN INTENSITY

Depression - PROMIS-29 SCORE

Symptom

Date Range

From - To Done

WITHIN NORMAL LIMITS	MILD	MODERTATE	SEVERE
20 30 40 50	60	70	80

Depression Score Trend Across Months of 2024

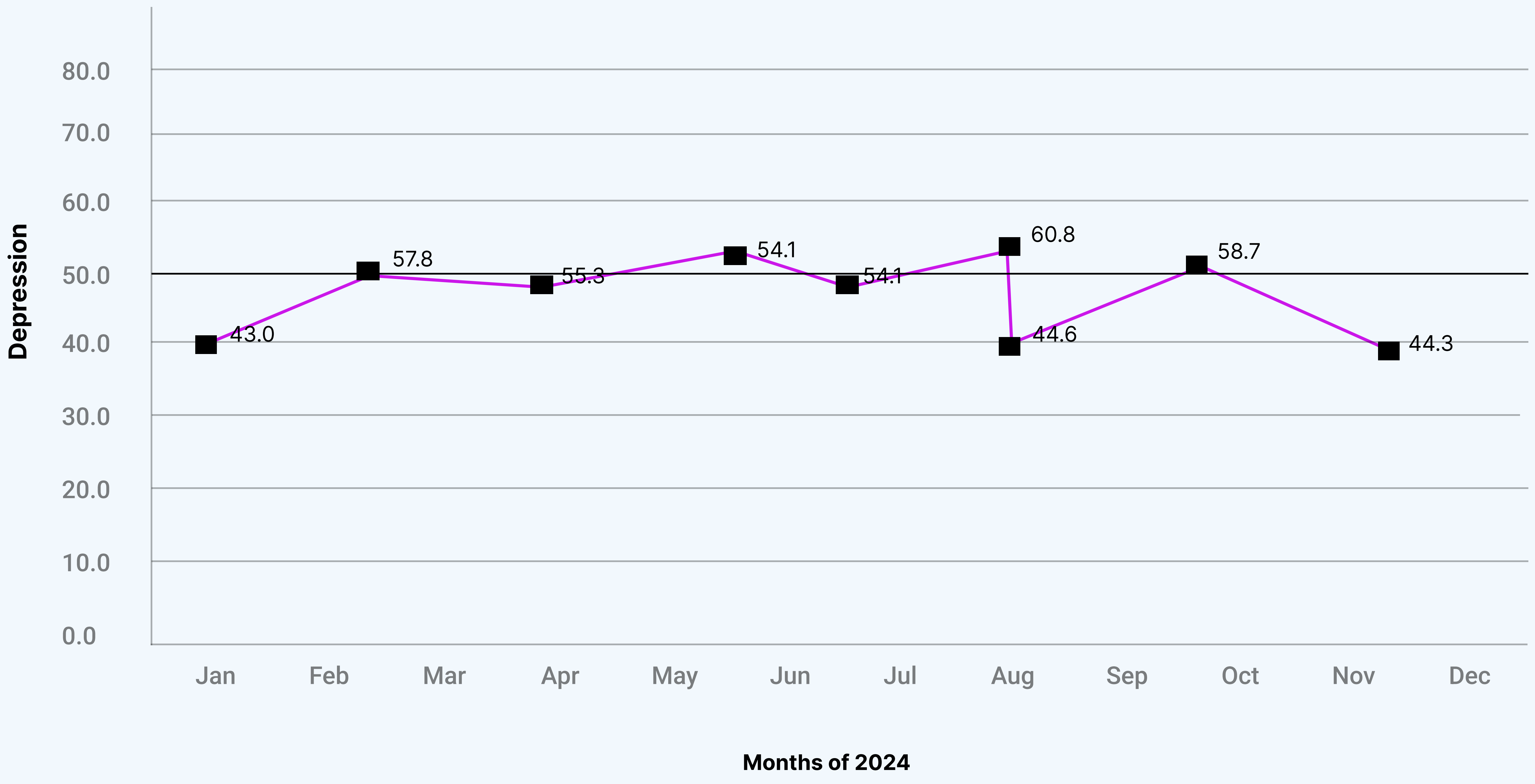

SMART Liver

Name: Jessica Taylor **MRN:** 65374683
Sex : Female **Age:** 45

LAB-BASED TASKS | CLINICAL PRACTICE GUIDELINES RECOMMENDATIONS | PATIENT REPORTED OUTCOME MEASURES (29) SCORE | PROGNOSIS SCORES

- ALL
- PHYSICAL FUNCTION
- ANXIETY
- DEPRESSION
- FATIGUE
- SLEEP DISTURBANCE
- SOCIAL PARTICIPATION
- PAIN INTERFERENCE
- PAIN INTENSITY

Depression - PROMIS-29 SCORE

Symptom

Date Range

From - To Done

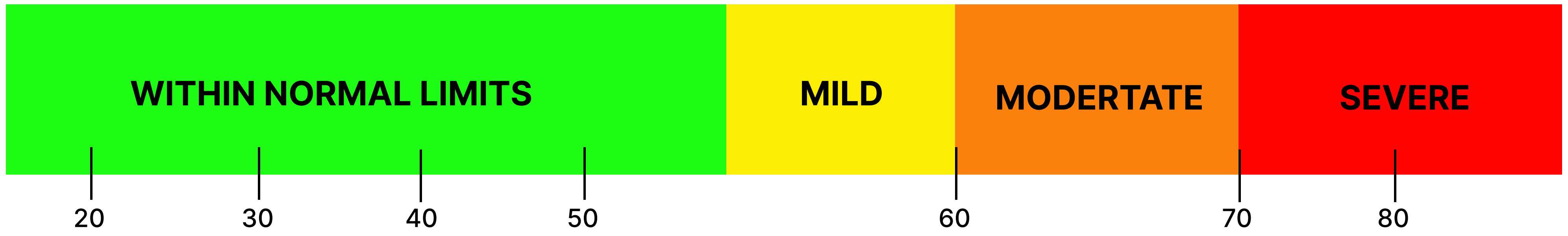

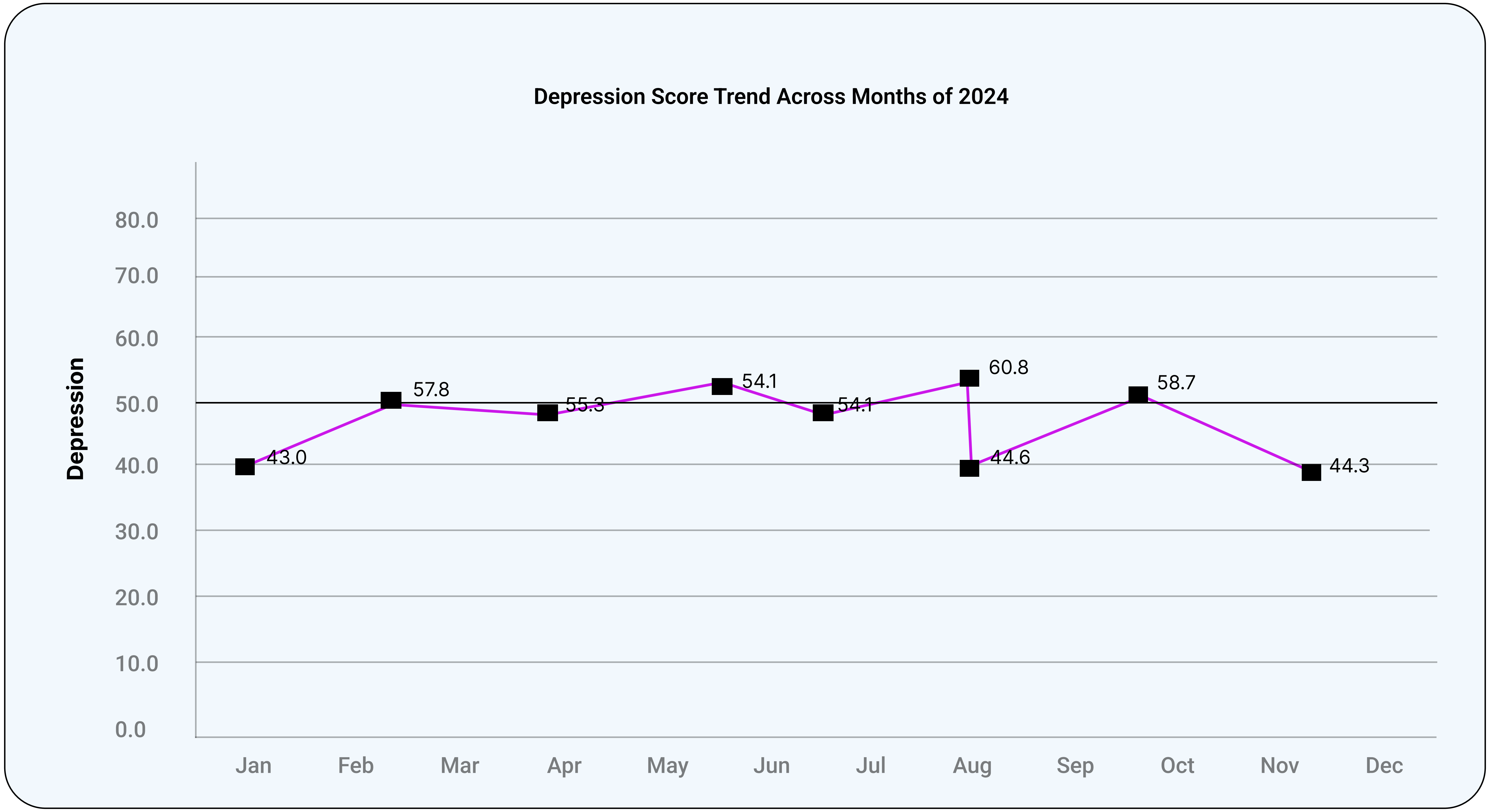

Months of 2024

SMART Liver

Name: Jessica Taylor **MRN:** 65374683
Sex : Female **Age:** 45

LAB-BASED TASKS | CLINICAL PRACTICE GUIDELINES RECOMMENDATIONS | PATIENT REPORTED OUTCOME MEASURES (29) SCORE | PROGNOSIS SCORES

- ALL
- PHYSICAL FUNCTION
- ANXIETY
- DEPRESSION
- **FATIGUE**
- SLEEP DISTURBANCE
- SOCIAL PARTICIPATION
- PAIN INTERFERENCE
- PAIN INTENSITY

Fatigue - PROMIS-29 SCORE

Symptom

Date Range
From - To Done

WITHIN NORMAL LIMITS	MILD	MODERTATE	SEVERE

20 30 40 50 60 70 80

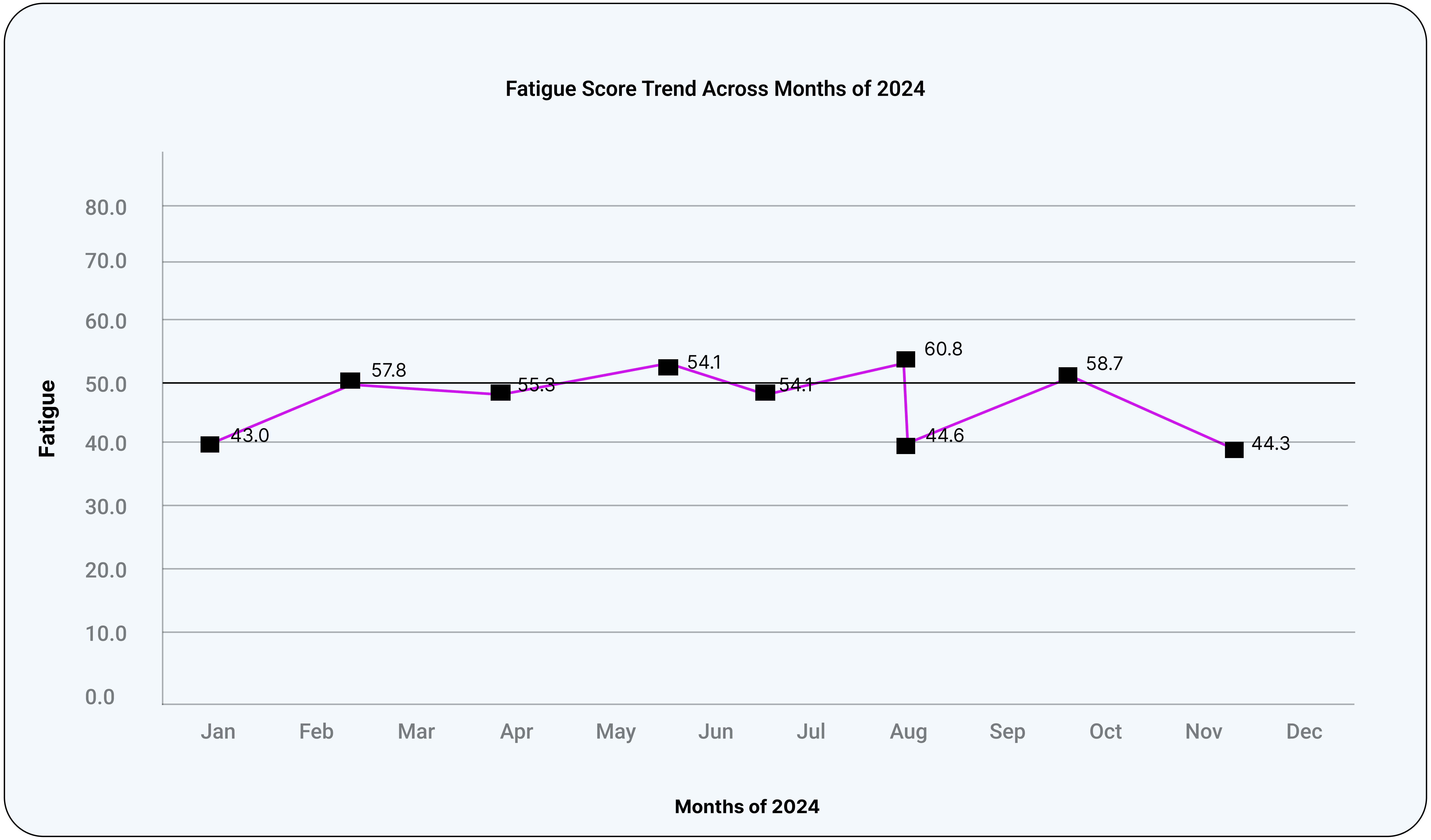

SMART Liver

Name: Jessica Taylor **MRN:** 65374683
Sex : Female **Age:** 45

LAB-BASED TASKS | CLINICAL PRACTICE GUIDELINES RECOMMENDATIONS | PATIENT REPORTED OUTCOME MEASURES (29) SCORE | PROGNOSIS SCORES

- ALL
- PHYSICAL FUNCTION
- ANXIETY
- DEPRESSION
- FATIGUE
- SLEEP DISTURBANCE
- SOCIAL PARTICIPATION
- PAIN INTERFERENCE
- PAIN INTENSITY

Sleep Disturbance - PROMIS-29 SCORE

Symptom

Date Range

From - To Done

WITHIN NORMAL LIMITS	MILD	MODERTATE	SEVERE
20 30 40 50	60	70	80

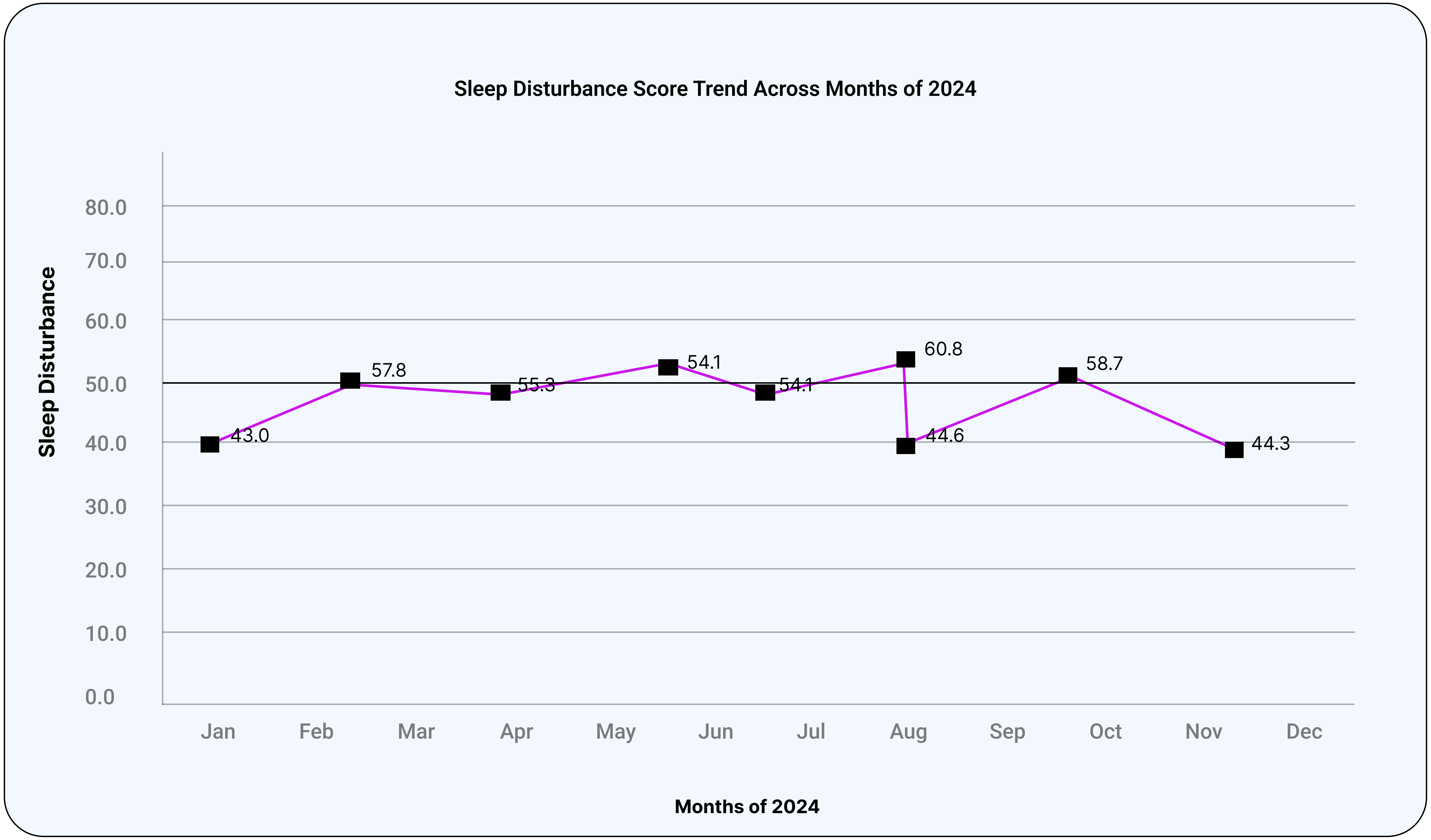

SMART Liver

Name: Jessica Taylor **MRN:** 65374683
Sex : Female **Age:** 45

LAB-BASED TASKS | CLINICAL PRACTICE GUIDELINES RECOMMENDATIONS | PATIENT REPORTED OUTCOME MEASURES (29) SCORE | PROGNOSIS SCORES

- ALL
- PHYSICAL FUNCTION
- ANXIETY
- DEPRESSION
- FATIGUE
- SLEEP DISTURBANCE
- SOCIAL PARTICIPATION
- PAIN INTERFERENCE
- PAIN INTENSITY

Social Participation - PROMIS-29 SCORE

Function

Date Range

From - To Done

WITHIN NORMAL LIMITS	MILD	MODERTATE	SEVERE
80 70 60 50	40	30	20

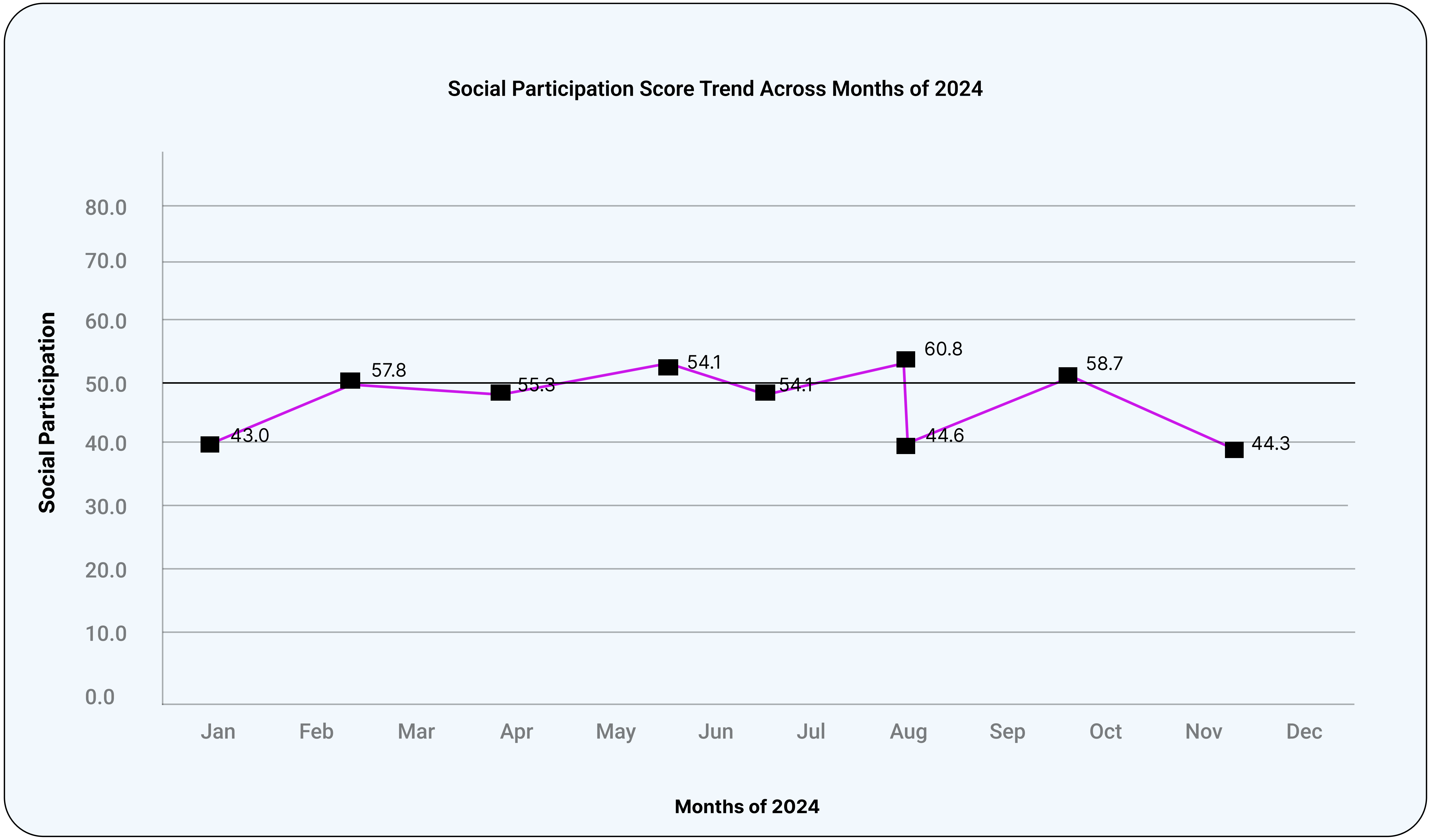

SMART Liver

Name: Jessica Taylor **MRN:** 65374683
Sex : Female **Age:** 45

LAB-BASED TASKS | CLINICAL PRACTICE GUIDELINES RECOMMENDATIONS | PATIENT REPORTED OUTCOME MEASURES (29) SCORE | PROGNOSIS SCORES

- ALL
- PHYSICAL FUNCTION
- ANXIETY
- DEPRESSION
- FATIGUE
- SLEEP DISTURBANCE
- SOCIAL PARTICIPATION
- PAIN INTERFERENCE
- PAIN INTENSITY

Pain Interference - PROMIS-29 SCORE

Symptom

Date Range

From - To Done

WITHIN NORMAL LIMITS	MILD	MODERTATE	SEVERE

20 30 40 50 60 70 80

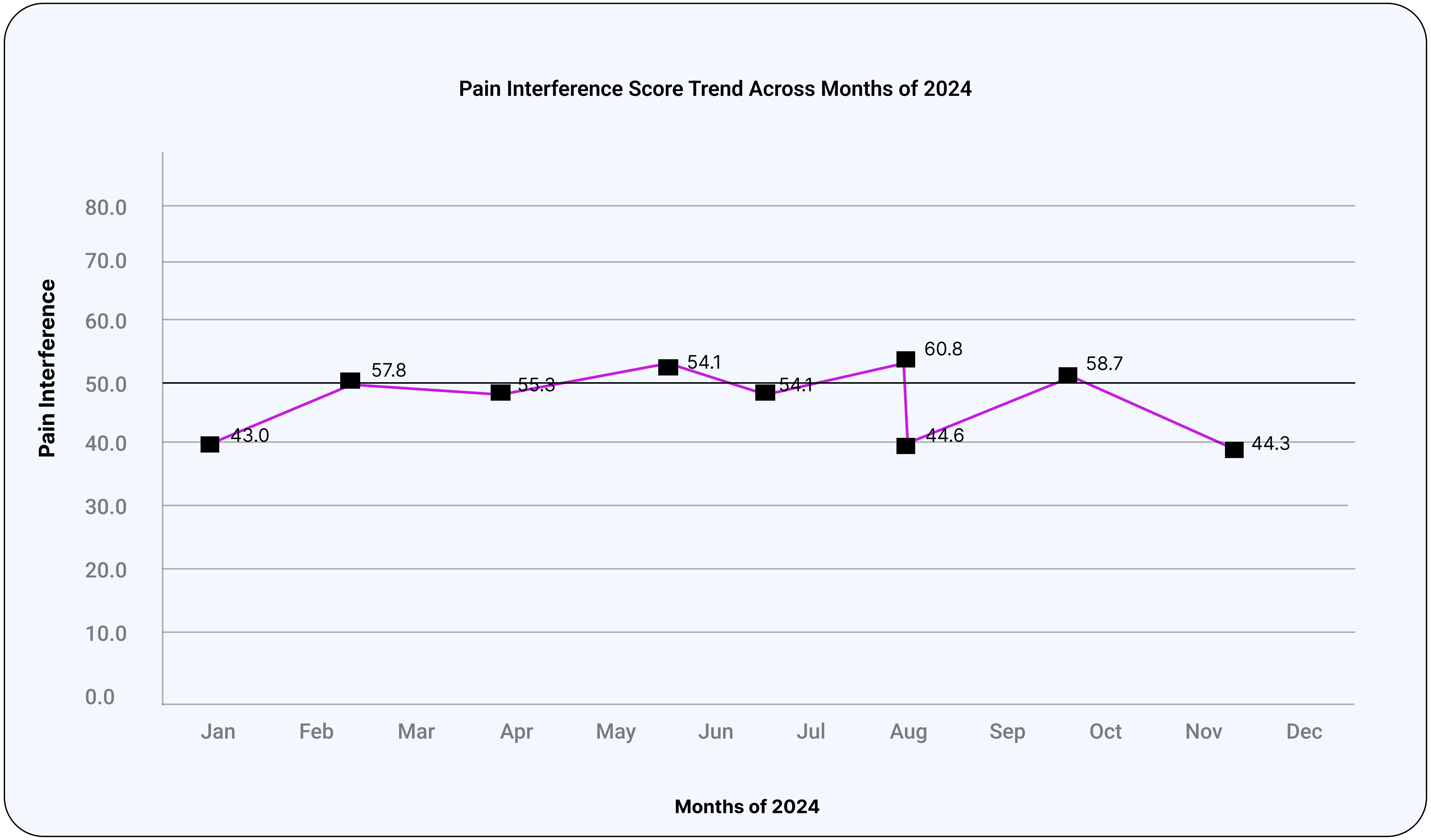

SMART Liver

Name: Jessica Taylor **MRN:** 65374683
Sex : Female **Age:** 45

LAB-BASED TASKS | CLINICAL PRACTICE GUIDELINES RECOMMENDATIONS | PATIENT REPORTED OUTCOME MEASURES (29) SCORE | PROGNOSIS SCORES

- ALL
- PHYSICAL FUNCTION
- ANXIETY
- DEPRESSION
- FATIGUE
- SLEEP DISTURBANCE
- SOCIAL PARTICIPATION
- PAIN INTERFERENCE
- PAIN INTENSITY

Pain Intensity - PROMIS-29 SCORE

Symptom

Date Range

From - To Done

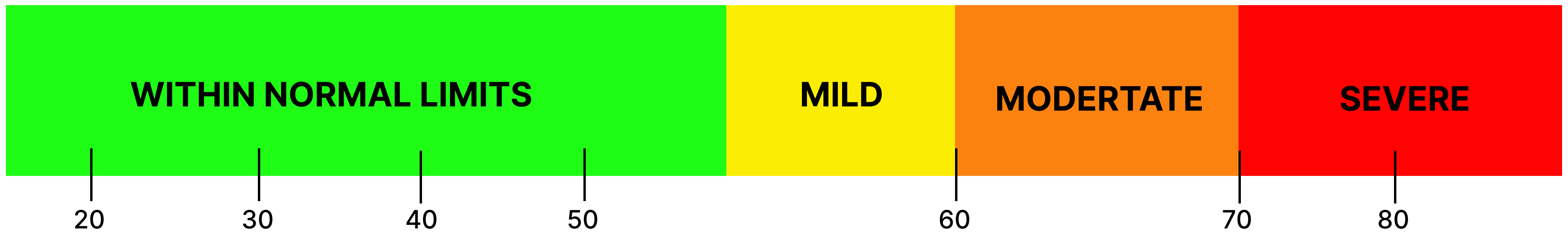

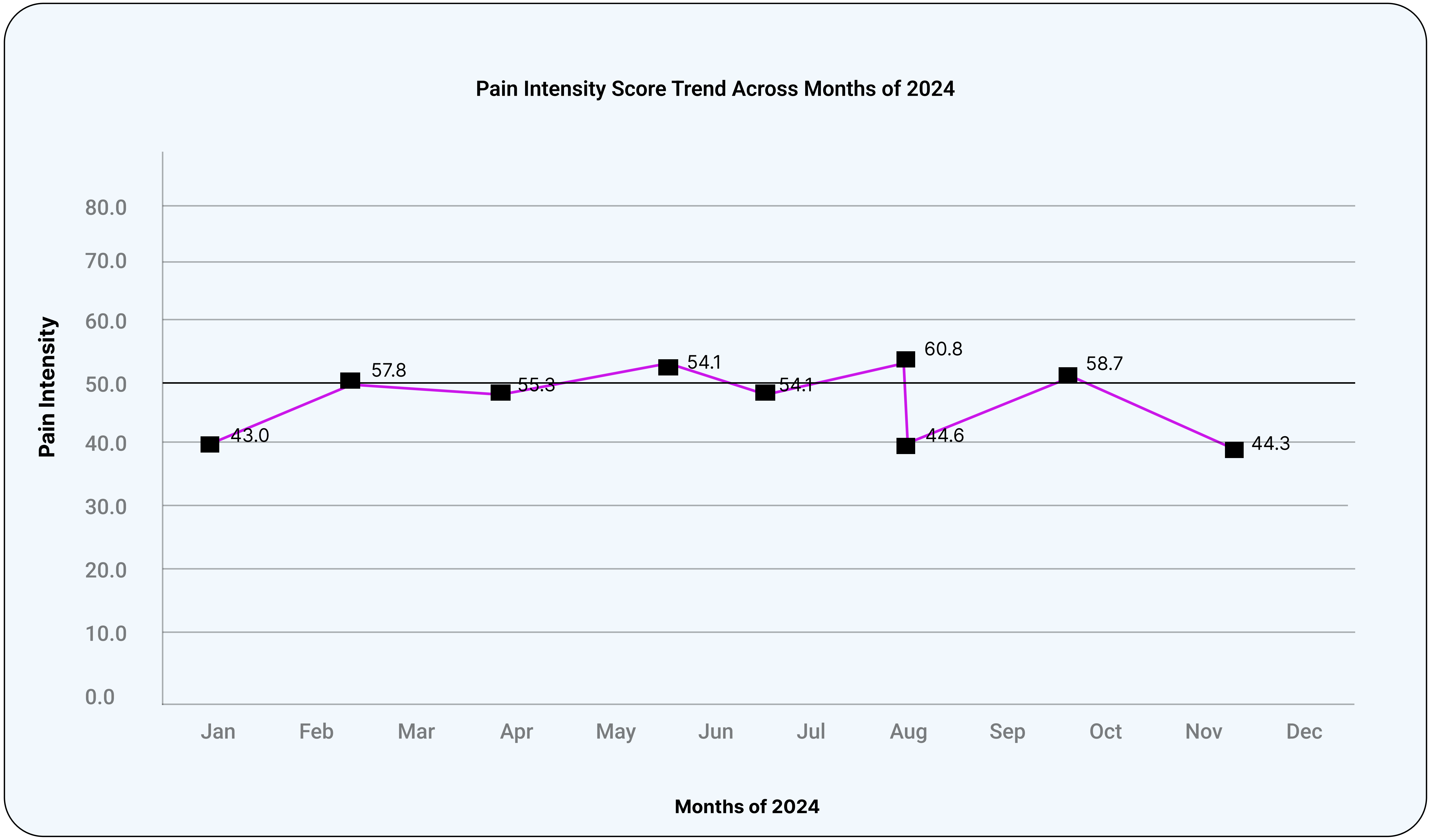

SMART Liver

Name: Jessica Taylor **MRN:** 65374683
Sex : Female **Age:** 45

LAB-BASED TASKS | CLINICAL PRACTICE GUIDELINES RECOMMENDATIONS | PATIENT REPORTED OUTCOME MEASURES (29) SCORE | PROGNOSIS SCORES

- ALL
- PHYSICAL FUNCTION
- ANXIETY
- DEPRESSION
- FATIGUE
- SLEEP DISTURBANCE
- SOCIAL PARTICIPATION
- PAIN INTERFERENCE
- COGNITIVE FUNCTION

Anxiety - PROMIS-29 SCORE

From mm/dd/yyyy **To** mm/dd/yyyy Done

Symptom

WITHIN NORMAL LIMITS	MILD	MODERATE	SEVERE

20 30 40 50 60 70 80

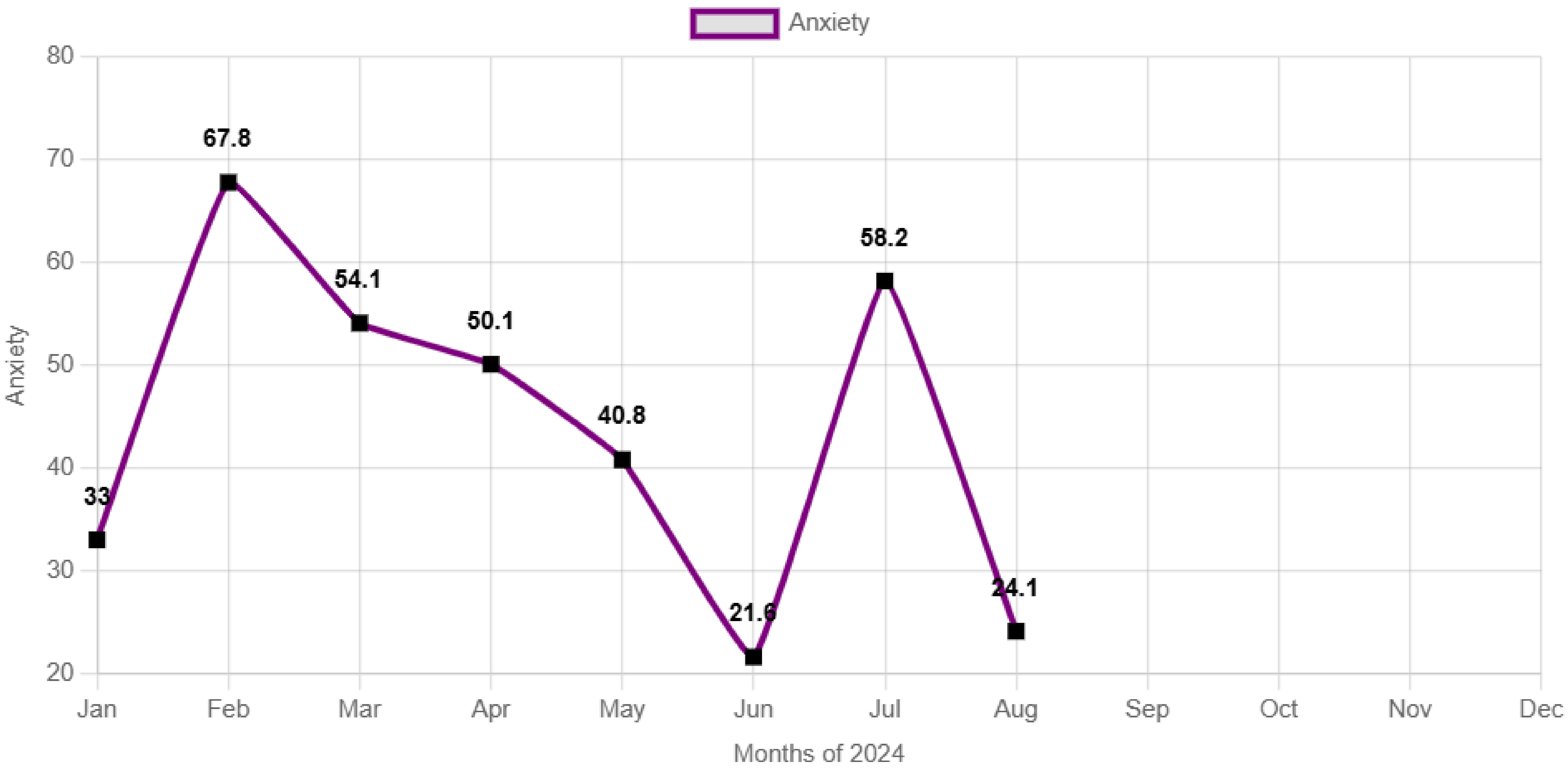

Supplement: S1 Fig — (PDF) [file pdig.0000969.s001.pdf]
