# Supplementary material for: A pilot feasibility study of human-centered design for cirrhosis care: Development and pilot testing of SMARTLiver prototype, a FHIR-based clinical decision support system for hepatology
Source: PLOS Digit Health. 2026 Jan 20;5(1):e0000969. doi: 10.1371/journal.pdig.0000969 (PMC12818595; doi:10.1371/journal.pdig.0000969)
Supplement: S1 Data — (DOCX) [file pdig.0000969.s002.docx]

Appendix 1: FHIR R4 Resources from Cerner for SMARTLiver

# **1. Introduction**

The SMARTLiver application leverages Cerner's (now Oracle Health) FHIR R4 API to integrate with electronic health records and facilitate team-based care coordination. This document describes the FHIR R4 resources utilized for patient data management, care planning, and clinical information exchange.

Cerner's FHIR R4 implementation follows the HL7 FHIR R4 (v4.0.1) specification and supports both open and secured endpoints with OAuth 2.0 and SMART on FHIR authentication protocols. The implementation is compliant with the US Core Implementation Guide v3.1.1 for interoperability requirements.

# **2. FHIR Resources Overview**

SMARTLiver utilizes the following categories of FHIR R4 resources from Cerner's Millennium Platform:

## **2.1 Base Resources**

### Patient Resource

**Endpoint:** /Patient

**Description:** Core demographic and administrative information about patients with chronic liver disease enrolled in the SMARTLiver program.

**Key Elements:**

- Demographics (name, birthDate, gender, address)
- Identifiers (MRN, SSN, insurance IDs)
- Contact information (phone, email)
- Language preferences
- Emergency contacts

### Practitioner Resource

**Endpoint:** /Practitioner

**Description:** Healthcare providers involved in patient care including hepatologists, nurses, and care coordinators.

**Key Elements:**

- Provider credentials and qualifications
- Specialty (hepatology, gastroenterology)
- Contact information and availability

### Organization Resource

**Endpoint:** /Organization

**Description:** Healthcare organizations and facilities providing care.

**Key Elements:**

- Hospital/clinic information
- Liver transplant centers
- Specialty departments

## **2.2 Clinical Resources**

### Condition Resource

**Endpoint:** /Condition

**Description:** Diagnoses and health conditions related to liver disease.

**Key Elements:**

- Primary diagnosis (cirrhosis, hepatitis, NASH)
- Comorbidities
- MELD/MELD-Na scores
- Child-Pugh classification
- ICD-10 codes

### Observation Resource

**Endpoint:** /Observation

**Description:** Clinical observations, lab results, and vital signs.

**Key Elements:**

- Liver function tests (ALT, AST, bilirubin, albumin)
- Coagulation studies (INR, PT)
- Vital signs (blood pressure, weight, BMI)
- Ascites assessment
- Hepatic encephalopathy grading

### AllergyIntolerance Resource

**Endpoint:** /AllergyIntolerance

**Description:** Patient allergies and intolerances affecting treatment.

**Key Elements:**

- Drug allergies
- Food intolerances
- Severity and reactions

## **2.3 Care Management Resources**

### CarePlan Resource

**Endpoint:** /CarePlan

**Description:** Coordinated care plans for liver disease management.

**Key Elements:**

- Treatment goals and objectives
- Planned activities and interventions
- Care team members
- Educational material assignments

### Goal Resource

**Endpoint:** /Goal

**Description:** Treatment goals and patient objectives.

**Key Elements:**

- Clinical targets (lab value goals)
- Lifestyle modification goals
- Medication adherence targets

### CareTeam Resource

**Endpoint:** /CareTeam

**Description:** Multidisciplinary care team composition.

**Key Elements:**

- Primary hepatologist
- Nurse coordinators
- Dietitians
- Social workers
- Transplant coordinators

## **2.4 Medication Resources**

### MedicationRequest Resource

**Endpoint:** /MedicationRequest

**Description:** Prescription orders for liver disease medications.

**Key Elements:**

- Diuretics (furosemide, spironolactone)
- Beta-blockers (propranolol)
- Lactulose
- Rifaximin
- Dosing instructions and refills

### Immunization Resource

**Endpoint:** /Immunization

**Description:** Immunization records critical for liver disease patients.

**Key Elements:**

- Hepatitis A and B vaccinations
- Pneumococcal vaccine
- Influenza vaccine
- COVID-19 vaccination status

## **2.5 Administrative Resources**

### Encounter Resource

**Endpoint:** /Encounter

**Description:** Clinical encounters and visits.

**Key Elements:**

- Outpatient visits
- Hospital admissions
- Telehealth consultations
- Emergency department visits

### Appointment Resource

**Endpoint:** /Appointment

**Description:** Scheduled appointments and follow-ups.

**Key Elements:**

- Clinic appointments
- Lab appointments
- Imaging studies
- Transplant evaluations

## **2.6 Documents and Reports**

### DocumentReference Resource

**Endpoint:** /DocumentReference

**Description:** Clinical documents and reports.

**Key Elements:**

- Progress notes
- Consultation reports
- Discharge summaries
- Educational materials

### DiagnosticReport Resource

**Endpoint:** /DiagnosticReport

**Description:** Diagnostic test reports and results.

**Key Elements:**

- Laboratory reports
- Imaging studies (ultrasound, CT, MRI)
- Endoscopy reports
- Liver biopsy results

### Procedure Resource

**Endpoint:** /Procedure

**Description:** Procedures performed on patients.

**Key Elements:**

- Paracentesis
- Variceal banding
- TIPS procedures
- Liver transplant

# **3. Implementation Details**

## **3.1 Authentication and Authorization**

The SMARTLiver application uses OAuth 2.0 with SMART on FHIR for secure authentication and authorization. The implementation supports:

- Standalone launch for patient-facing mobile app
- EHR launch for provider-facing web application
- Backend services for automated data synchronization
- Refresh tokens for maintaining session continuity

## **3.2 API Endpoints**

**Open Sandbox:**

https://fhir-open.cerner.com/r4/{tenantId}/{resource}

**Secure Endpoints:**

Non-Patient: https://fhir-ehr-code.cerner.com/r4/{tenantId}/{resource}

Patient Access: https://fhir-myrecord.cerner.com/r4/{tenantId}/{resource}

## **3.3 Data Exchange Format**

All data exchanges use JSON format with FHIR content-type:

**Content-Type:** application/fhir+json

## **3.4 Search Parameters**

Common search parameters supported across resources:

- _id: Resource identifier
- patient: Patient reference
- date: Date ranges for temporal queries
- category: Resource categorization
- _count: Pagination control

# **4. Compliance and Standards**

The implementation adheres to the following standards and regulations:

- HL7 FHIR R4 v4.0.1 specification
- US Core Implementation Guide v3.1.1
- SMART on FHIR v2.1 specifications
- 21st Century Cures Act requirements
- HIPAA Privacy and Security Rules
- ONC Health IT Certification requirements
