# Supplementary material for: A pilot feasibility study of human-centered design for cirrhosis care: Development and pilot testing of SMARTLiver prototype, a FHIR-based clinical decision support system for hepatology
Source: PLOS Digit Health. 2026 Jan 20;5(1):e0000969. doi: 10.1371/journal.pdig.0000969 (PMC12818595; doi:10.1371/journal.pdig.0000969)
Supplement: S2 Data — (DOCX) [file pdig.0000969.s003.docx]

**S2 Data: Firebase and FCM Implementation for Team-Based Care Workflow in SMARTLiver Application**

This supplementary document describes the technical implementation of the Firebase and Google Firebase Cloud Messaging (FCM) infrastructure used in the SMARTLiver application to enable real-time collaboration and task management between healthcare providers and patients. The implementation supports the team-based care workflow shown in the application's user interface, facilitating educational material distribution, task assignment, and care coordination.

# **2. System Architecture Overview**

## **2.1 Technology Stack**

The SMARTLiver application utilizes a hybrid cloud architecture combining:

- **Google Cloud Firestore:** HIPAA-compliant NoSQL database for storing non-PHI collaborative data
- **Firebase Cloud Functions:** Serverless functions for business logic and data processing
- **Firebase Cloud Storage:** Secure storage for educational materials and documents
- **Firebase Realtime Database:** Real-time synchronization for task status updates
- **Google Identity Platform:** HIPAA-compliant authentication service

## **2.2 Data Architecture**

The system implements a segregated data architecture to ensure HIPAA compliance:

- **PHI Data:** Stored in Cerner EHR via FHIR APIs
- **Collaborative Data:** Task assignments, status updates stored in Firestore
- **Educational Content:** Static materials in Cloud Storage
- **Real-time Updates:** Task status synchronization via Realtime Database

# **3. Data Models and Collections**

## **3.1 Firestore Collections**

### Tasks Collection

Collection: /tasks

{

taskId: string,

patientId: string,

taskName: string,

dueDate: timestamp,

status: 'overdue' | 'upcoming' | 'completed',

assignedTo: {

recipientId: string,

recipientType: 'patient' | 'provider' | 'nurse' | 'MA',

recipientName: string

},

createdBy: string,

createdAt: timestamp,

completedAt: timestamp | null,

category: string,

}

### Educational Materials Collection

Collection: /educational_materials

{

materialId: string,

category: string,

subcategory: string,

title: string,

contentUrl: string,

contentType: 'video' | 'pdf' | 'article' | 'interactive',

assignedPatients: string[],

viewCount: number,

lastUpdated: timestamp

}

### Care Team Collection

Collection: /care_teams

{

teamId: string,

patientId: string,

members: [

{

memberId: string,

role: string,

name: string,

permissions: string[]

}

],

primaryProvider: string

}

# **4. Implementation Features**

## **4.1 Educational Materials Management**

The system provides comprehensive educational content management:

| **Category** | **Content Types** | **Delivery Method** |
| --- | --- | --- |
| ESLD Complications | Ascites, Varices, HE, Pleural Effusion | Video, PDF, Interactive |
| Procedures & Treatments | Ablation, Embolization, Lab Tests, Liver Biopsy | Video tutorials, Step guides |
| Healthy Living | Advance Care Planning, Alcohol, Cannabis, Care Partner Support | Articles, Worksheets |
| Symptom Relief | Anxiety, Depression, Itching, Muscle Cramps, Pain | Self-help guides, Videos |

## **4.2 Task Management System**

The task management system enables providers to assign and track tasks across the care team:

### Task Assignment Workflow

1. Provider creates task through web interface
2. Task stored in Firestore with real-time sync
3. Push notification sent to assigned recipient
4. Recipient receives task in mobile app (Carepal)
5. Status updates sync across all devices
6. Completion triggers care team notification

### Task Status Management

Tasks are automatically categorized by status:

- **Overdue:** Tasks past due date requiring immediate attention
- **Upcoming:** Tasks scheduled within the next 7 days
- **Completed:** Finished tasks for audit and review

## **4.3 Cloud Functions Implementation**

Key serverless functions managing the workflow:

### Task Assignment Function

exports.assignTask = functions.firestore

.document('tasks/{taskId}')

.onCreate(async (snap, context) => {

const task = snap.data();

// Send notification to recipient

// Update care team dashboard

// Log activity in audit trail

});

### Status Update Function

exports.updateTaskStatus = functions.firestore

.document('tasks/{taskId}')

.onUpdate(async (change, context) => {

const newStatus = change.after.data().status;

const oldStatus = change.before.data().status;

// Sync with FHIR CarePlan if needed

// Update real-time dashboard

});

# **5. Security and Privacy Implementation**

## **5.1 HIPAA Compliance Strategy**

The implementation ensures HIPAA compliance through:

- **Data Segregation:** PHI stored only in Cerner EHR, never in Firebase
- **Reference Architecture:** Tasks reference patient IDs without containing PHI
- **Encryption:** All data encrypted at rest (AES-256) and in transit (TLS 1.3)
- **Access Control:** Role-based permissions enforced via Security Rules
- **Audit Logging:** All access and modifications logged for compliance

## **5.2 Firestore Security Rules**

rules_version = '2';

service cloud.firestore {

match /databases/{database}/documents {

// Tasks collection

match /tasks/{taskId} {

allow read: if isCareTeamMember();

allow write: if isProvider();

allow update: if isAssignee();

}

}

}

## **5.3 Authentication Flow**

1. User authenticates via Google Identity Platform
2. JWT token generated with custom claims for role
3. Token verified by Firebase Security Rules
4. Access granted based on role and care team membership
5. Session maintained with refresh tokens

# **6. Real-time Collaboration Features**

## **6.1 Live Task Updates**

The system provides real-time synchronization across all connected devices:

- WebSocket connections for instant updates
- Offline capability with sync on reconnection
- Conflict resolution for concurrent edits
- Optimistic UI updates for better user experience

## **6.2 Message Broadcasting**

Providers can send messages to selected recipients or the entire care team:

- Secure messaging without PHI exposure
- Push notifications to mobile devices
- Read receipts and delivery confirmation
- Message history with search capability

# **7. Integration with FHIR Resources**

## **7.1 Bidirectional Sync**

The Firebase system maintains synchronization with FHIR resources:

| **Firebase Collection** | **FHIR Resource** | **Sync Direction** |
| --- | --- | --- |
| /tasks | CarePlan.activity | Bidirectional |
| /care_teams | CareTeam | FHIR → Firebase |
| /educational_materials | DocumentReference | Firebase → FHIR |
| /symptom_reports | Observation | Firebase → FHIR |

# **8. Performance and Scalability**

## **8.1 Performance Optimizations**

- Composite indexes for complex queries
- Pagination for large data sets
- Caching frequently accessed data
- CDN for educational content delivery
- Lazy loading of non-critical resources

## **8.2 Scalability Architecture**

- Auto-scaling Cloud Functions
- Multi-region deployment for global access
- Database sharding for large datasets
- Load balancing across instances

# **9. Monitoring and Analytics**

## **9.1 System Monitoring**

- Real-time performance dashboards
- Error tracking and alerting
- Usage analytics and patterns
- Compliance audit trails

## **9.2 Clinical Analytics**

- Task completion rates by role
- Educational material engagement metrics
- Care team collaboration patterns
- Patient outcome correlations

# **10. Future Enhancements**

Planned improvements to the Firebase/Firecloud implementation:

- AI-powered task prioritization
- Natural language processing for symptom reports
- Machine learning for care plan optimization
- Predictive analytics for risk stratification
- Voice-enabled task management
