# Supplementary material for: A pilot feasibility study of human-centered design for cirrhosis care: Development and pilot testing of SMARTLiver prototype, a FHIR-based clinical decision support system for hepatology
Source: PLOS Digit Health. 2026 Jan 20;5(1):e0000969. doi: 10.1371/journal.pdig.0000969 (PMC12818595; doi:10.1371/journal.pdig.0000969)
Supplement: Attachment — Submitted filename: SmartLiver-Response_to_reviewers.docx [file pdig.0000969.s005.docx]

Oct 19, 2025

**RESPONSE TO REVIEWERS**

We thank the reviewers for their thoughtful and constructive comments, which have substantially improved our manuscript. We have carefully addressed each comment with comprehensive revisions. All changes are tracked in the revised manuscript, and we provide detailed responses below.

**Reviewer #1**

We appreciate the reviewer's recognition of our work's significance, particularly noting the importance of our Cerner-based implementation. We have addressed all nine comments as follows:

**Comment 1: Focus introduction on fragmented data as the major challenge**

**Response:** We agree completely and have refocused the introduction to immediately identify data fragmentation as the primary barrier to effective cirrhosis care. The opening paragraph now begins: "Fragmented clinical data represents the most significant barrier to effective chronic liver disease management, forcing clinicians to navigate multiple disconnected electronic health record (EHR) modules to gather essential patient information." We emphasize throughout that providers must synthesize data across 6-8 different screens, directly linking this fragmentation to low guideline adherence rates.

**Comment 2: Remove sections on EHR history and information blocking**

**Response:** We have removed both sections entirely ("The Electronic Health Record Fragmentation Crisis" and "Controversies and Ongoing Challenges"). In their place, we added a brief transitional paragraph that directly connects the fragmentation problem to our SMART-on-FHIR solution, maintaining narrative flow while eliminating the unnecessary historical context.

**Comment 3: Note structured methods in Research Aims section and add HCD diagram**

**Response:** We have added a paragraph in the Research Aims section explicitly noting that "Our methodology employed an iterative human-centered design process, including clinical workflow observation, stakeholder interviews, prototype development, and usability testing with healthcare providers (detailed methods are presented at the end of this manuscript)." We have also created Figure 2, a comprehensive Human-Centered Design Process Diagram showing our five-phase methodology from clinical discovery through usability evaluation.

**Comment 4: Add method summaries to Results subsections**

**Response:** We have added one-sentence method summaries at the beginning of each Results subsection:

- **Human-Centered Design Process:** "We conducted full-day observations of hepatology providers at Indiana University Health, systematically documenting their Cerner EHR navigation patterns and workflow challenges for cirrhosis management."
- **Quantitative Usability Results:** "We administered the validated Health-ITUES survey to five clinical team members (2 RNs, 1 MA, 1 PA, 1 physician) following structured demonstrations of SMARTLiver's features."
- **Qualitative Feedback Analysis:** "Using Think-Aloud protocols during task completion and affinity mapping of recorded feedback, we identified recurring usability themes across participant roles."

**Comment 5: Reorder Likert scale colors and add figure legend**

**Response:** We have created an updated Figure 3 with properly ordered Likert scale colors (Strongly Disagree to Strongly Agree: dark red, light red, gray, light green, dark green) and added a comprehensive figure caption: "Figure 3. Health-ITUES Usability Survey Results by Domain. Stacked bar chart showing participant responses (n=5) across five usability domains. Response categories follow the Likert scale from Strongly Disagree (1) to Strongly Agree (5)."

**Comment 6: Clarify the 73% calculation**

**Response:** We have clarified this confusing percentage by providing the actual numbers: "Among the five participants, workflow integration questions received mixed responses: on the primary integration item, 2 participants (40%) strongly agreed, 2 participants (40%) agreed, and 1 participant (20%) remained neutral that the application integrated well into clinical workflows. When averaging across all Integration & Workflow survey items, the positive response rate was 80% (combining 'agree' and 'strongly agree' responses)."

**Comment 7: Address data completeness limitations**

**Response:** We have added a new paragraph "Data Completeness Considerations" in the Limitations section acknowledging that SMARTLiver's reliance on structured FHIR data elements may not capture all clinically relevant information. We explicitly note that "Laboratory results, clinical observations, and other critical data often exist in free-text notes, scanned documents from external providers, or PDF attachments that fall outside the current application's extraction capabilities." We suggest future iterations should explore natural language processing and optical character recognition technologies.

**Comment 8: Highlight Cerner-based development**

**Response:** We have added prominent text emphasizing this contribution: "Notably, our development and testing occurred entirely within a Cerner-based EHR environment at Indiana University Health, marking a significant departure from the predominant focus on Epic implementations in SMART-on-FHIR and human-centered design literature." We also added discussion noting this "demonstrates the framework's vendor-agnostic potential while highlighting the additional complexity required for non-Epic implementations."

**Comment 9: Address IU Health's transition to Epic**

**Response:** We have added a section "Implementation Continuity Considerations" in Future Research Directions discussing the 2027 transition to Epic. We frame this as "a unique opportunity to validate SMARTLiver's cross-vendor portability—a critical test of SMART-on-FHIR's 'write once, run anywhere' promise." We outline two RCT options: (1) accelerate trial completion within Cerner before transition, or (2) design the trial with a planned interim analysis coinciding with the EHR migration to assess performance across both platforms.

## Reviewer #2

We thank the reviewer for recognizing the novelty of our SMART-on-FHIR approach to addressing fragmented patient information. We have addressed both comments:

### Comment 1: Claims should match the limited usability data

**Response:** We have comprehensively revised the manuscript to temper all claims and align them with our pilot study design and small sample size (n=5). Key changes include:

- **Title:** Now reads "A pilot feasibility study of human-centered design for cirrhosis care"
- **Abstract:** Changed "successfully aggregated" to "demonstrated technical feasibility" and added "though clinical effectiveness remains to be demonstrated"
- **Discussion:** Opening now acknowledges "preliminary usability testing with five participants offers early insights" and states "larger studies are needed to validate these observations"
- **Throughout:** We consistently use tentative language ("may," "suggests," "preliminary," "initial evidence") rather than definitive statements
- **Removed:** Broad generalizations such as "workflow integration trumps technical interoperability" replaced with "workflow integration may be an important consideration alongside technical interoperability, though broader evaluation is needed"

### Comment 2: Add supplementary information for rebuilding the logic

**Response:** We appreciate this suggestion and have included detailed technical specifications in as Supplementary docs, including:

- FHIR resource mappings and integration details
- Links to UI design files in Figma
- Firebase Cloud Messaging and Firestore implementation

We would be happy to provide additional supplementary materials including code repositories or detailed API documentation if the journal supports such supplements.

## Reviewer #3

We appreciate the reviewer's recognition that our work addresses a well-recognized problem and provides valuable insights about clinician preferences for data aggregation over algorithmic sophistication.

### Comment 1: Reframe as prototype at proof-of-concept stage

**Response:** We have systematically reframed the entire manuscript to position SMARTLiver as a proof-of-concept prototype:

- **Throughout manuscript:** Consistently use "prototype" instead of "application" or "tool"
- **Discussion opening:** Now states "This pilot study provides initial evidence that SMART on FHIR technology may help address EHR fragmentation"
- **Three core findings:** All rewritten with tentative language acknowledging limitations
- **Problematic statement removed:** "workflow integration trumps technical interoperability" replaced with balanced, tentative observation
- **Conclusions:** Now explicitly states "The SMARTLiver prototype demonstrates proof-of-concept" and "clinical effectiveness and broader adoption remain to be evaluated through larger studies"

### Comment 2: Adjust title to indicate pilot/feasibility study

**Response:** We have revised the title to: "A pilot feasibility study of human-centered design for cirrhosis care: Development and pilot testing of SMARTLiver prototype, a FHIR-based clinical decision support system for hepatology." The title now clearly indicates this is a pilot feasibility study and emphasizes the prototype nature of the system.
